# Country-Specific External Costs of Abiotic Resource Use Based on User Cost Model in Life Cycle Impact Assessment

**DOI:** 10.1021/acs.est.4c00100

**Published:** 2024-04-26

**Authors:** Ryosuke Yokoi, Masaharu Motoshita, Takeshi Matsuda, Norihiro Itsubo

**Affiliations:** † Research Institute of Science for Safety and Sustainability, National Institute of Advanced Industrial Science and Technology (AIST), 16-1 Onogawa, Tsukuba 305-8569, Japan; ‡ Pacific Power Co., Ltd., 3-22 Kandanishikicho, Chiyoda, Tokyo 101-0054, Japan; § Faculty of Science and Engineering, Waseda University, 3-4-1 Okubo Shinjuku-ku, Tokyo 169-8050, Japan

**Keywords:** life cycle assessment, characterization, metal, mineral resource, scarcity, availability, externality, economic evaluation

## Abstract

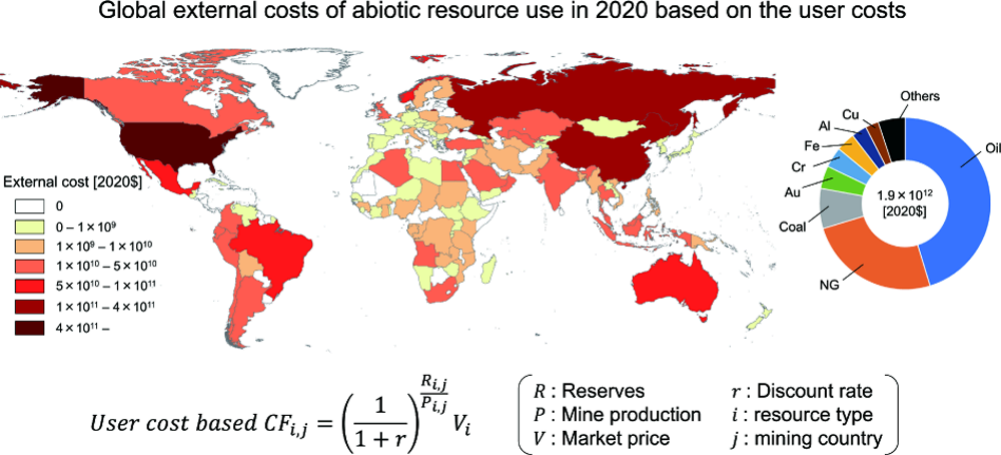

Abiotic resources are indispensable in society, but there are concerns regarding their depletion, scarcity, and increasing prices, resulting in potential economic damage in the future. To address these concerns, it is effective to consider the external costs of resource use. Although resource availability is different among mining sites, and local conditions are relevant in assessing resource scarcity, previous studies have assessed external costs and potential impacts of abiotic resource use globally. This study provides country-specific characterization factors (CFs) of abiotic resource use in life cycle impact assessment based on the user cost model, which represents the external costs of abiotic resource use to reflect country-specific resource scarcity. We demonstrate considerable variations in the CFs depending on the mining country, suggesting that the choice of mining country can affect external costs. The global external cost of abiotic resource use in 2020 was estimated at 1.9 trillion $, with a major contribution from the extraction of fossil fuels in the United States. Historical trends of the CFs and relevant parameters showed temporal fluctuations, emphasizing the importance of regularly updating the data underlying the calculation of the CFs. Country-level assessments of the external costs of resource use can contribute to discussions on the responsibilities of consuming countries by incorporating material footprint studies.

## Abstract 2

We present country-specific external costs of abiotic resource use based on the user cost model. We highlight the importance of the country-specific and temporal contexts in the assessment of the externality of current abiotic resource use for future generations.

## 1 Introduction

Abiotic resources play a crucial role in economic development and the advancement of low-carbon technologies. However, due to their finite nature and uneven distribution, there are concerns regarding the scarcity of their geological stocks, which can lead to reduced accessibility, supply shortages, and price increases, ultimately causing economic damage in the future. Even if they will not be depleted globally for the time being, mining activity may stop in a specific region because of the increase in scarcity in the region, which leads to a mining country concentration and reduced accessibility. Mining country concentration is relevant to a lowered resilience in material supply chains and higher supply risks.^1,2^ Although such issues may require the development of new or previously uneconomic orebodies, developing new mining tends to face high risks in environmental, social, and governance contexts.^3−6^ To ensure sustainable resource use and economic development, it is important to consider the future implications of abiotic resource use when we currently use abiotic resources. One way to address this damage is by considering the external costs of abiotic resource use and reflecting their depletion in current prices.^7^

In life cycle assessment (LCA), the concerns associated with abiotic resource use are addressed as potential impacts of abiotic resource use under the area of protection “natural resources” in life cycle impact assessment (LCIA).^8^ The safeguard subject for mineral resources has been proposed as follows:^9^ “potential to make use of the value that mineral resources can hold for humans in the technosphere.” The damage due to mineral resource use is quantified as the reduction or loss of this potential. As reviewed by Alvarenga et al.,^10^ Berger et al.,^9^ Klinglmair et al.,^11^ and Sonderegger et al.,^12^ various methods have been developed to assess the potential impacts of abiotic resource use in LCIA, which evaluate different aspects associated with abiotic resource use in impact pathways. For example, the depletion methods, including abiotic depletion potential (ADP),^13^ assess a reduction of the availability of abiotic resources (i.e., depletion); the future efforts methods, including surplus ore potential and surplus cost potential,^14,15^ focus on the consequences of current abiotic resource use on future efforts to extract additional abiotic resources; and the thermodynamic accounting methods, including cumulative exergy extraction from the natural environment (CEENE),^16^ quantify the cumulative exergy (or energy) used in a product system. In addition, methods addressing a dissipation concept (flows to sinks or stocks that are not accessible to future users) have been recently developed.^17−19^ These methods do not focus on the extraction of geological stocks but on the dilution of the total stocks. Among the methods assessing abiotic resource use in LCIA, two methods explicitly focus on the external costs of abiotic resource use: future welfare loss^20^ and user cost.^21^

Future welfare loss is based on Hotelling’s rule, which posits that the economic rent (i.e., the difference between resource price and extraction cost) of an exhaustible resource will increase at the same rate as the market interest rate.^22^ It is also based on the theory that the social discount rate is generally lower than the market discount rate.^20^ Following Hotelling’s rule, the resource price of the last unit sold at depletion can be calculated using the market discount rate, market price, extraction cost, and depletion time. The socially optimal price can then be retroactively determined by using the social discount rate. Because the social discount rate is lower than the market discount rate, the retroactively determined socially optimal price is higher than the current market price. The difference between the socially optimal price and the current market price is defined as the future welfare loss.^20^

The user cost was employed to calculate the characterization factors (CFs) of abiotic resource use for an end point in LIME2 on a global scale and then updated in LIME3 to provide country-specific CFs, which are LCIA methods developed in Japan.^21,23^ It is based on El Serafy’s user cost concept, which proposes setting aside a portion of the earnings from the sale of resources for capital investment to generate a perpetual stream of income.^24^ The user cost concept assesses the external cost of resource use from the perspective of future miners. In the “task force mineral resources” of the “global guidance for LCIA indicators and methods” project led by the Life Cycle Initiative under the auspices of UN Environment, the user cost model adopted in LIME2 is among the recommended methods for evaluating the potential impacts of mineral resource use in LCIA, which addresses the question “how can I quantify the relative (economic) externalities of mineral resource use?”^9^ While some characterization models, such as ADP, surplus ore potential, surplus cost potential, and CEENE, focus on global-scale impacts of abiotic resource use, the user cost model addresses concerns related to mining by the current generation leading to future resource scarcity at specific mining sites as well as global-scale. Although abiotic resources are traded worldwide and their availability is a global issue, stopped mining and external costs for future miners are country-level issues. The balance between extraction rate and resource availability (i.e., pressure on mining capacity) differs among mining sites, and thus, local conditions are relevant in assessing resource scarcity and the potential impacts of mining on future generations.^25,26^ Therefore, country-specific CFs for abiotic resource use can improve the assessment of the potential impacts of abiotic resource use. However, country-specific CFs for abiotic resource use have not yet been published in peer-reviewed studies but have only been proposed in the gray literature.^21^

This study presents regionalized CF at the country level based on the user cost model applied to an extraction flow as an elementary flow in LCA, which represents the country-specific external costs of abiotic resource use. We aim to explore the characteristics and relevance of these country-specific CFs based on the user cost. To do so, we demonstrate the variation in country-specific CFs and their historical trends, along with the relevant parameters influencing the CFs. Furthermore, the global external cost of abiotic resource use is estimated using the user cost model to evaluate its significance in the context of the externality of current global resource use. Additionally, a comparison with other characterization models is presented to illustrate the effect of selecting different characterization models on the LCIA results. The target year is 2020 and the CFs are calculated for 29 mineral resources and three fossil fuels, which are chosen to cover major abiotic resources under the limited data availability: aluminum (Al), antimony (Sb), barium (Ba), boron (B), chromium (Cr), cobalt (Co), copper (Cu), fluorine (F), gold (Au), iron (Fe), lead (Pb), lithium (Li), magnesium (Mg), manganese (Mn), molybdenum (Mo), nickel (Ni), niobium (Nb), palladium (Pd), phosphorus (P), platinum (Pt), rhenium (Re), silver (Ag), tantalum (Ta), tin (Sn), titanium (Ti), tungsten (W), uranium (U), vanadium(V), zinc (Zn), coal, natural gas (NG), and oil.

## 2 Methods

### 2.1 User Cost Model

**Figure 1:**
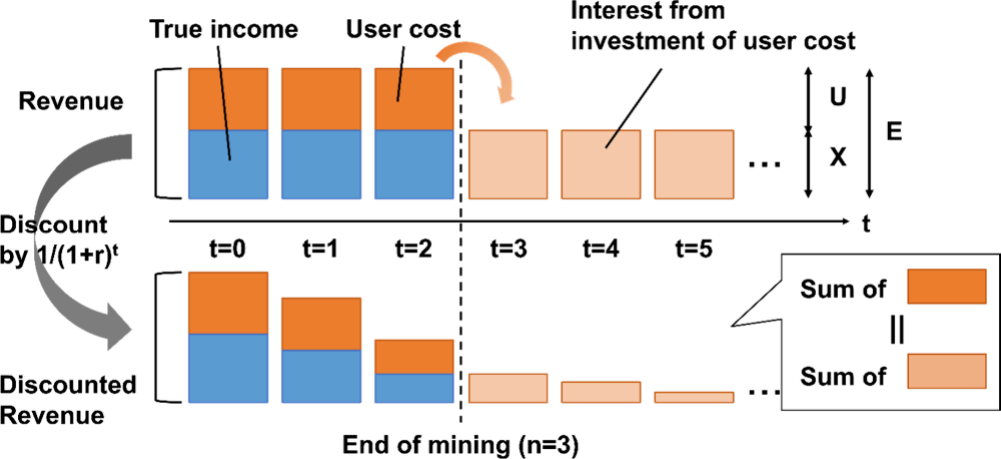
Concept of the user cost. The dark and light orange bars represent the user cost and interest from the investment of user cost after the mine closure, respectively. The blue bar represents the true income before mine closure when a constant income is sustained and is equal to the interest from the investment of user cost before discount (the light orange bar in the upper row). The sum of discounted user costs until the end of mining is equal to the sum of discounted constant income after the end of mining.

El Serafy^24^ presented user costs as the investment needed to sustain a constant income after a mine is closed. The “user cost” refers to a cost that users would incur due to the use of capital assets, and in this study, the “users” are future miners of abiotic resources. Figure 1 illustrates a conceptual diagram of user costs. The earnings from the sale of resources (*E*) are divided into two portions: user cost (*U*) and constant income (*X*). To ensure a constant income both during mining as well as after mine closure, a portion of the earnings (i.e., user cost) needs to be set aside every year and invested. Taking into account the discount rate (*r*), the sum of user costs during mining is equal to the sum of constant income after mine closure:
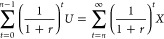
1where *n* is the number of years remaining for mining (years) (hereinafter called the depletion time). The user cost can then be expressed using eq 2.
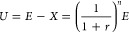
2The depletion time (*n*) is estimated by dividing reserves by annual mine production, and earnings from the sale of resources (*E*) are calculated by multiplying mine production by the market price:
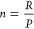
3
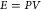
4where *R* is the reserves (kg), *P* is the mine production (kg/year), and *V* is the market price of refined metals or fossil fuels ($/kg).

### 2.2 Country-Specific CFs Based on the User Cost

One feature of user costs is that country-specific external costs can be derived based on country-specific data. The country-specific analysis can reflect the differences in resource availability and mining-related situations among countries and can contribute to the discussion on changes in mining sites and supply chain management.^27^ The country-specific user cost for mining abiotic resource *i* in country *j* in year *t* is calculated by using eq 5.
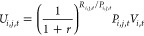
5Country-specific CFs based on user costs, which represent the external costs of mining a unit of abiotic resource in each country, were calculated using eq 6.
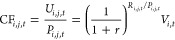
6where CF_*i*,*j*,*t*_ is the country-specific CF based on the user cost of mining abiotic resource *i* in country *j* in year *t*. When the mining countries are unknown, evaluators can adopt the weighted average of the CFs by country-specific mine production (WCF_*i*,*t*_), which is calculated using eq 7.
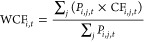
7

### 2.3 Data

Data for country-specific mine production and reserves in 2020 were obtained from the USGS,^28^ except for uranium, coal, natural gas, and oil, which were obtained from the NEA^29^ and Energy Institute^30^ (previously published by BP). Regarding the three fossil fuels, “coal” includes commercial solid fuels (i.e., bituminous coal and anthracite (hard coal), lignite and brown (sub-bituminous) coal, and other commercial solid fuels), “natural gas” represents gas (i.e., not liquefied natural gas), and “oil” includes crude oil, shale oil, oil sands, condensates, and natural gas liquids, according to the Energy Institute.^30^ While different options are available for natural stock estimates of mineral resources, such as resources, reserve base, and ultimate reserves,^28^ we adopted “reserves” for reasons of availability for updated and country-specific data and the number of covered resources. Reserves represent natural stocks that could be economically extracted at the time of determination.^28^ However, there is criticism that reserves are not suitable for long-term assessment of resource availability because they are influenced by various factors, such as price and technologies, and can fluctuate over time.^31−33^ The choice of natural stock estimates in characterization models is often discussed for the depletion methods and is also an issue for the user cost model. Limitations of the use of reserves in the assessment of user costs of resource use will be discussed in the Section 4. Regarding uranium, the identified recoverable resources (cost range is < USD 130/kgU) presented by the NEA were used for natural stock estimates. In addition, we adopted the proved (1P) reserves for oil obtained from the Energy Institute, while they are also controversial, and the use of oil consultancy proved-plus-probable (2P) reserves has been proposed.^34^ The estimates of reserves used for calculating the user cost of oil are also one limitation of this study and should be tackled in the future. Data on historical market prices were obtained, and the five-year average prices were calculated to limit the influence of short-term market volatility. The data sources used for this analysis include the Energy Institute,^30^ Trading Economics,^35^ USGS,^36^ and USGS.^37^ Data sources of mine production, reserves, and market price for each abiotic resource are summarized in Table S1. We used a discount rate of 3% as the default. Additionally, historical data on mine production, reserves, and market prices from 2000 to 2020 were collected to analyze the trends in user costs, market prices, and depletion times over time. The market prices for each year were converted to dollars in 2020 (2020$) using the Consumer Price Index. Data for calculating the CFs (i.e., mine production, reserves, and price) are shown in the Supplementary Excel File.

## 3 Results

### 3.1 Country-Specific CFs Based on the User Cost

**Figure 2:**
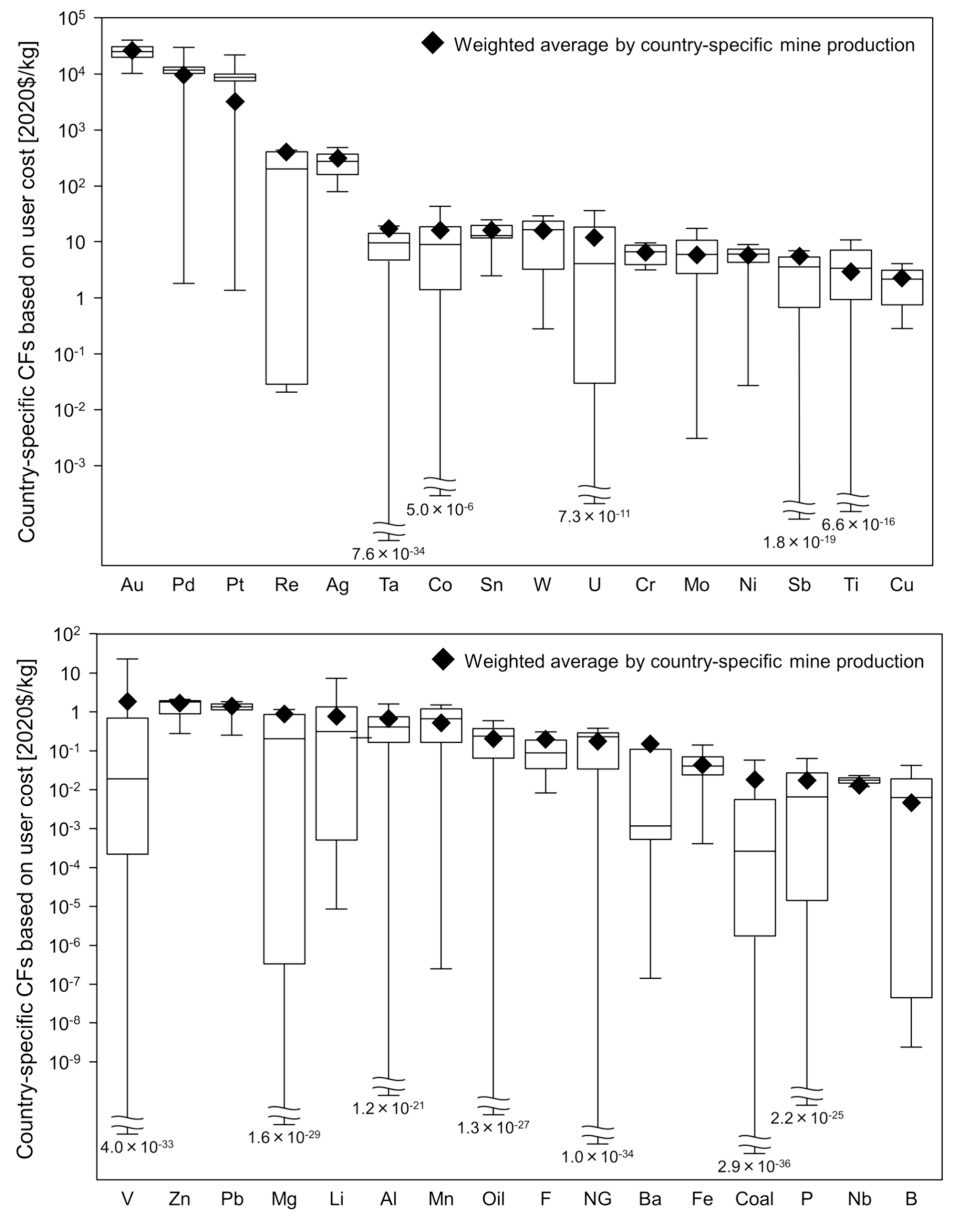
Country-specific characterization factors (CFs) based on user cost of resources in 2020 in the descending order of weighted average by mine production. A logarithmic scale is used for the vertical axis.

Country-specific CFs based on user costs for 32 resources in 2020 are presented in Figure 2, arranged in the descending order of the weighted average of CFs by country-specific mine production (WCF). The CF lists for all countries (193 countries are covered) are shown in theSupplementary Excel File. In cases in which data on mine production or reserves were not available for a specific country, the WCF was adopted. It is noteworthy that certain resources exhibited substantial variations in CFs depending on the mining country, owing to differences in the depletion time. This highlights the significant effect that the choice of the mining country can have on the external costs of resource use. Most of the resources with high values of WCF were precious metals: Au (2.7 × 10^4^ $/kg), Pd (9.9 × 10^3^ $/kg), Pt (3.3 × 10^3^ $/kg), and Ag (3.2 × 10^2^ $/kg). These values are more than 2 orders of magnitude larger than those of common metals, such as Al (6.7 × 10^–1^ $/kg), Cu (2.3 $/kg), and Fe (4.5 × 10^–2^ $/kg).

**Figure 3:**
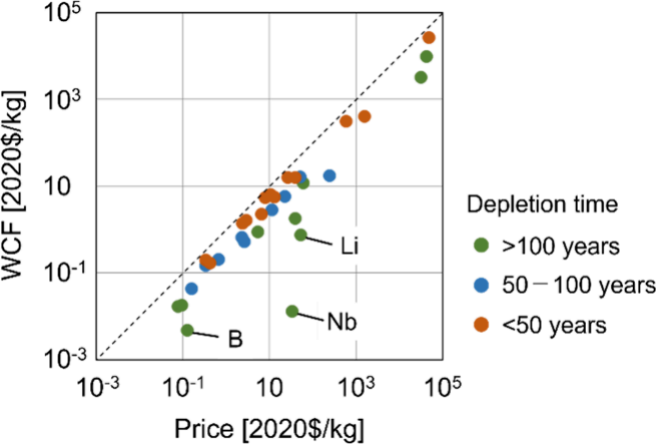
Relationship between the weighted average of CFs and the price of resources in 2020. The colors of the plots represent the categories of the depletion time of resources. WCF: weighted average of characterization factors.

The CFs based on user costs demonstrated a strong correlation with market prices (Figure 3). The top five resources in the WCF (Au, Pd, Pt, Re, and Ag) coincided with market prices. Meanwhile, some resources, including B, Li, and Nb, exhibited relatively low WCF values compared with the market prices. This is primarily attributed to the long depletion times of these resources. Figure 3 suggests that resources with relatively long depletion times showed relatively low CFs compared with the market prices, as a long depletion time results in a lower user cost, as shown in eq 2. Furthermore, the CFs for mining countries with large mine production had a significant effect on the WCF. The relationship between mine production and the CFs for each mining country is depicted in Figure S1. When countries with large mine production demonstrated lower CFs (i.e., large reserves compared to mine production), the WCF tended to be relatively low compared to the market price, as observed for abiotic resources such as B, Li, and Nb. The effects of these parameters on country-specific CFs are discussed in the Section 4.

### 3.2 Global External Costs of Resource Use Based on the User Cost

**Figure 4:**
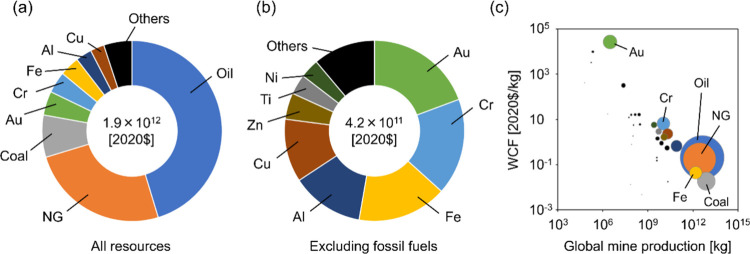
Share of global external costs of resource use based on user cost in 2020. (a) All target abiotic resources. (b) Target abiotic resources excluding fossil fuels. (c) Relationships between global mine production and WCF of resources. The size of the circles represents the global external costs (i.e., the product of global mine production and WCF). The colors of the circles correspond to those in (a) and (b).

The sum of the products of country-specific CFs and mine production for all countries provides the annual global external cost of resource use (Figure 4). In 2020, the global external cost of resource use amounted to 1.9 × 10^12^ $, which accounted for approximately 2.2% of the world GDP. This value is comparable to the overall environmental costs generated by global resource extraction (mainly attributed to climate change), which were estimated to be 3.5 × 10^11^ and 4.4 × 10^12^ $ for the low and maximum estimates, respectively.^38^ The distribution of external costs among resources indicates that a small number of resources accounted for the majority of the external costs (Figure 4a,b). The three primary contributors to external costs were fossil fuels: oil (45.3% of global external costs), natural gas (25.0%), and coal (7.5%). Among mineral resources, gold (4.3%), chromium (3.8%), iron (3.6%), aluminum (2.9%), and copper (2.5%) had relatively high external costs. The high external costs of these resources can be attributed to their large mine production, except for gold, which had a small mine production but high CFs (Figure 4c). Chromium had substantial values for both mine production and CF, resulting in high external costs. On the other hand, in terms of global environmental costs of resource extraction estimated by Arendt et al.,^38^ iron had the largest contribution (19%), followed by coal (16%), magnesium (11%), manganese (11%), and natural gas (10%), which showed different trends from our results for external costs of abiotic resource use in terms of the user cost.

The geographical distribution of external costs of resource use is depicted in Figure S2, highlighting significant variations among mining countries. The United States accounted for a substantial portion of the global external costs of all resources (25.3%), followed by China (16.0%), Russia (8.4%), Brazil (4.5%), and Australia (4.1%). Fossil fuels had a dominant share in these countries, particularly in the United States, Russia, and Brazil (Figures S3 and S4). The high external costs in the United States were primarily attributed to its significant share of mine production and high country-specific CFs for oil and natural gas (Figure S5). Although Russia did not exhibit high country-specific CFs, it had a relatively large share of the mine production for these fossil fuels. Focusing on mineral resources, a distinct geographical distribution of external costs was evident (Figure S2). China accounted for the highest external costs for mineral resources (25.0% of global external costs), followed by Australia (11.2%), South Africa (8.9%), Turkey (6.3%), and India (4.5%). The primary mineral resources contributing to these external costs varied among these countries: aluminum for China, iron for Australia and India, and chromium for South Africa and Turkey (Figures S3 and S4).

Country-specific analysis of external costs can help identify countries with significant external costs of resource use. However, it should be noted that extracted resources are not consumed but accumulated in society as in-use stocks, which can be used in the future. Consideration of in-use stocks and dilution of total stocks (i.e., dissipation) will be discussed in Section 4.3. In addition, the external costs of resource use are induced by the final demands of consuming countries, whereas we focus on the external costs attributed to mining in producing countries in this study. Future studies should consider analyzing the consumption-based external costs of resource use to provide a more comprehensive understanding of the external costs.

### 3.3 Temporal Variations of the User Costs and Relevant Parameters

**Figure 5:**
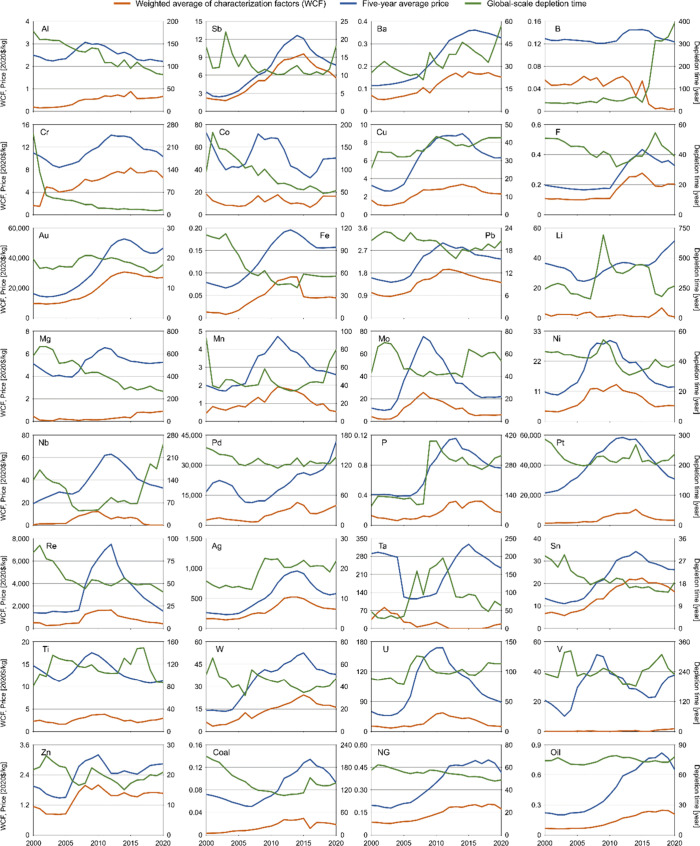
Historical trends of the weighted average of characterization factors based on user cost (WCF) (left axis), five-year average price (left axis), and global-scale depletion time (right axis). Global-scale depletion time was calculated by using global mine production and reserves.

Market prices and depletion time, the main parameters determining user cost, exhibited considerable temporal fluctuations, resulting in corresponding fluctuations in the WCF (Figure 5). These fluctuations also led to changes in the order of WCF, although the top five resources with the highest WCF remained consistent throughout (Figure S6). The resources with the largest fluctuations in WCF (i.e., the highest coefficient of variation) were V, Ta, Nb, Mg, and Li (Figure S7). The historical trends of the WCF generally aligned with the trends in market prices for most resources, particularly for Sb, Ba, Cu, F, Au, Pb, Mo, Ni, Re, Ag, Sn, W, Zn, natural gas, and oil (Table S2). However, for certain resources, such as Al, B, Li, Mg, Ta, and V, the historical trends in market prices had a lesser effect on the WCF. This can be attributed to the relatively stable prices for these resources, leading to a stronger influence on the historical trends in depletion time. However, it should be noted that V exhibited a unique pattern, where its historical trends in both market price and depletion time showed a weaker correlation with the WCF. The unique behavior of V can be explained by its limited number of mining countries (five countries were reported in 2020 according to USGS^28^). Any changes in the depletion time in a country with significant mine production had a substantial effect on the WCF. In the case of V, fluctuations in the depletion time in Brazil had a particularly significant effect on the WCF, resulting in the largest fluctuations observed. These findings emphasize the importance of regularly updating the data underlying the calculation of CFs to account for changes in the depletion time and other relevant parameters.

**Figure 6:**
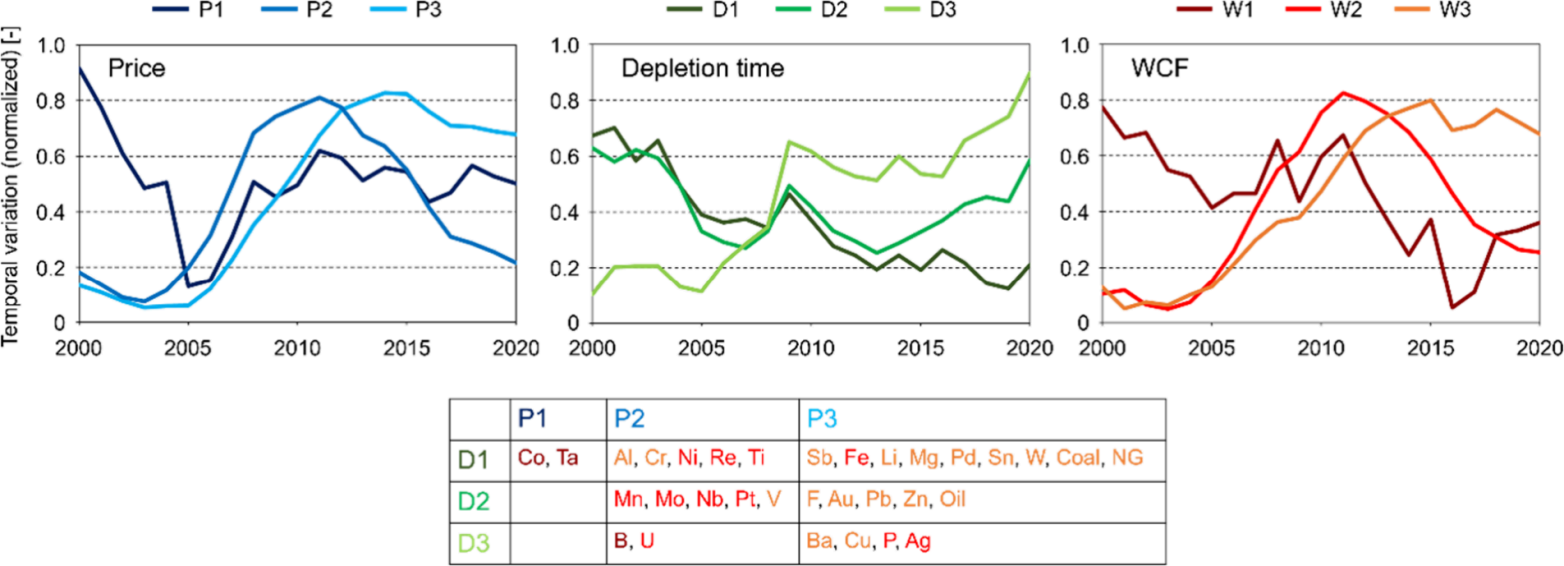
Average historical trends of the price, depletion time, and WCF for each cluster. Historical trends are normalized in the range 0–1. The colors of characters for resources in the table correspond to those for clusters of the WCF in the graph.

To understand the general historical trends of user cost and relevant parameters, the target resources were classified using time-series clustering (k-means clustering) of the historical data for market price, depletion time, and WCF^39^ (Table S3). There were three clusters (P1, P2, and P3 for market price; D1, D2, and D3 for depletion time; and W1, W2, and W3 for WCF). The normalized average historical trends for each cluster for each parameter demonstrated similarities in trends between the market price and the WCF (Figure 6). The classifications of resources based on the clustering of market prices and WCF were similar for most resources (P1, P2, and P3 correspond to W1, W2, and W3, respectively). For both clusters, the first cluster (P1 and W1) showed a decreasing and then an increasing trend, the second cluster (P2 and W2) showed an increasing and then a decreasing trend, and the third cluster (P3 and W3) showed an increasing and then a stable trend. Meanwhile, the clustering of the depletion time and WCF showed different results (Figures 6 and S8). The ratio of minimum and maximum values in the normalized average historical trends during 2000–2020 were largest for WCF (14.3–16.5 times), followed by market price (6.9–15.4 times) and depletion time (2.5–8.7 times) (Table S4). These results indicate that CFs based on user cost generally exhibit temporal fluctuations and have different trends among resources, suggesting that the choice of target year may have a significant effect on the LCIA results. These trends were also observed in other characterization models based on mine production and natural stock estimates (e.g., ADP).^40^ If evaluators aim to mitigate fluctuations in CFs and their effects on specific resources, depending on the target year, then adopting the moving average of CFs for several years is recommended. Given these fluctuations associated with underlying data, the relevance of using the user cost and how to use this model in LCIA should be further discussed by analyzing the characteristics and conducting case studies in the future.

Temporal variations in user costs, combined with changes in mine production, have resulted in changes in the global external costs of abiotic resource use over time (Figure S9). The global external costs of resource use have increased, reaching a peak of 2.3 × 10^12^ $ in 2018, which is more than 4.5 times the value in 2000, and then decreased from 2018 to 2020. A decomposition analysis of changes in global external costs revealed that these changes were primarily driven by changes in WCF rather than changes in mine production^41^ (Figure S10). While changes in mine production have contributed to an increase in global external costs for most years, decreases in mine production for certain resources, such as Cr, Au, Coal, NG, and Oil, have led to a decrease in global external costs in 2020 (Figure S11).

## 4 Discussion

### 4.1 Comparison with Other Models

**Figure 7:**
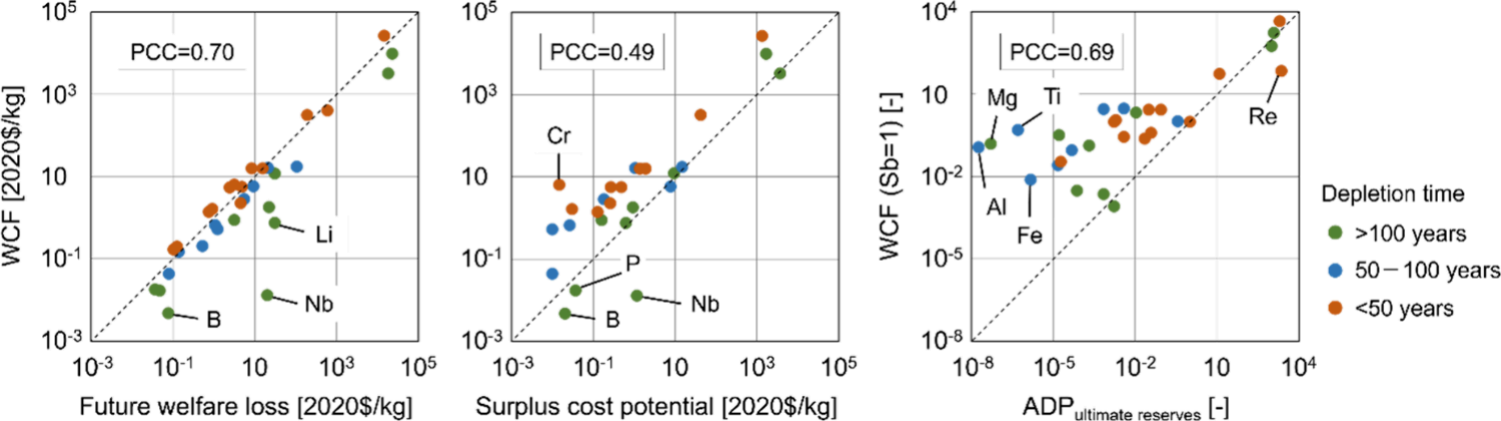
Comparison of different characterization models of resource use. The Pearson correlation coefficient (PCC) is shown in each scatter diagram.

Various characterization models have been proposed for abiotic resource use in LCIA. The WCF based on user cost was compared with other characterization models, including future welfare loss, surplus cost potential, and abiotic depletion potential based on ultimate reserves (ADP_ultimate reserves_) (Figure 7). Future welfare loss is another characterization model that assesses the external costs of abiotic resource use.^20^ While future welfare loss is calculated based on either resource-specific depletion time or a constant depletion time for all resources (300 years), the characterization factors based on resource-specific depletion time are compared with the WCF based on user cost. Surplus cost potential quantifies the impact of abiotic resource use in monetary units, focusing on the average increase in extraction cost caused by a decrease in ore grade.^15^ ADP_ultimate reserves_ is calculated by dividing annual mine production by the square of ultimate reserves and is represented as a value relative to a reference resource (Sb).^13,40,42^ It does not quantify the impacts of abiotic resource use in monetary units but is included in the comparison because it is a widely used characterization model for abiotic resource use. For the comparison with the ADP_ultimate reserves_ in Figure 7, the WCF based on the user cost was converted to the relative value of Sb to maintain consistency with the ADP_ultimate reserves_. A list of the characterization factors for these models is provided in the Supplementary Excel File.

Because both user costs and future welfare losses are calculated based on market prices, they show similar trends (Figure 7). However, the user cost is calculated to be larger when the depletion time is shorter, whereas the future welfare loss has the opposite characteristic. Therefore, resources with long depletion times, such as B, Li, and Nb, exhibit larger values based on future welfare losses. The surplus cost potential is represented in monetary units; however, the underlying concept differs from that of the user cost, resulting in different trends for CFs (Figure 7). In general, the surplus cost potential exhibits smaller values than the user cost, whereas some resources with long depletion times, including B, Nb, and P, show smaller characterization factors based on the user cost. This is because the surplus cost potential focuses on the increase in extraction cost and is not directly related to the depletion time. The ADP_ultimate reserves_ relative to Sb for most resources showed smaller values than the user cost relative to Sb, suggesting that the impact of Sb extraction was assessed as relatively significant based on the ADP_ultimate reserves_ compared to the user cost model (Figure 7). Because ultimate reserves have a significant effect on the calculation of ADP_ultimate reserves_, resources with large ultimate reserves, such as Al, Fe, Mg, Ti, and V, show considerably smaller values of ADP_ultimate reserves_ compared to the user cost (Figure S12).

These results suggest that different characterization models, even those represented in monetary units, can provide different results. There are various characterization models for abiotic resource use, which are different in mid- or end-point, assuming problems, and time perspectives.^43^ Therefore, it is essential to understand the underlying concepts and explore the differences between the characterization models and the effects of the selection of models. Recently, efforts have been made to develop guidance or frameworks for choosing a relevant model that is consistent with the perspectives of users to assess the impacts of abiotic resource use.^9,12,43,44^ Given the diversity of perspectives on abiotic resources and assessment models, choosing a relevant model according to the perspective while recognizing the dependency of results on used models would be one option.^45^ If other models with a similar perspective are available (e.g., user cost and future welfare loss), then sensitivity analysis using the other models will be effective. Furthermore, a project trying to develop a global consensus LCIA method, including characterization models for natural resources, is ongoing.^46^ Our results and discussion will provide insight into these activities.

### 4.2 Effects of Parameters on the CFs Based on the User Cost

**Figure 8:**
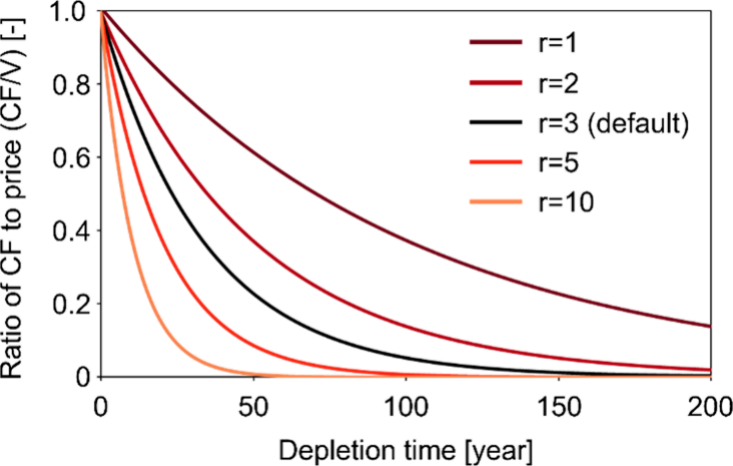
Effects of the Depletion Time and Discount Rate (*r*) on the CF to price ratio (CF/V).

The CF based on the user cost is determined by the depletion time, market price, and discount rate. The effects of changes in the depletion time and discount rate on the ratio of CF to the market price are shown in Figure 8. Although the market price directly affects the user cost, Figure 8 suggests that the depletion time has a significant effect on the CF. For example, in the case of a discount rate of 3% (default), the CF of a resource with a depletion time of 50 years is more than four times larger than that of the resource with a depletion time of 100 years. As mentioned in the Section 2 and also shown in Figure 5, reserves, mine production, and the resulting depletion time exhibit temporal fluctuations, leading to temporal fluctuations in the WCF. These results suggest the importance of updating underlying data for calculating user costs.

The discount rate also affects the CF, as suggested by other characterization models.^47^ The discount rate determines the effect of the difference in the depletion time on CF, and CF is calculated to be smaller with a higher discount rate. For example, the ratios of the CFs with 50 and 100 years of depletion time significantly differ depending on the discount rates: the ratios of the CFs are 1.6, 2.7, 4.4, 11.5, and 117.4 for discount rates of 1, 2, 3, 5, and 10%, respectively. Since the depletion time varied depending on the resources (Figure S13), the choice of discount rate affects the order of CFs and thus changes the main resources in terms of external costs (Figure S14). For example, Figure S14 shows that Cr is more significant in the global external costs calculated with a discount rate of 10% compared to the results for other discount rates because resources with a short depletion time are assessed as relatively significant with a higher discount rate based on user cost (Figure 8).

Although defining the appropriate or agreed-upon discount rate is challenging, we propose using a discount rate of 3% as the default, which is used in the Green Book for the social discount rate.^20,48^ Discount rate of 3% was also adopted in the surplus cost as default, which is another characterization model of ReCiPe 2008.^47^ Nevertheless, uncertainty related to the discount rate is one of the challenges of the user cost model as well as other assessment models considering the discount rate. Therefore, it is desirable to conduct a sensitivity analysis with different discount rates when adopting CFs based on user cost, even though exploring the appropriate discount rate is a significant task.

### 4.3 Limitations and Future Work

The user cost calculation does not require a large amount of data. It can be calculated using the depletion time, which is estimated as the ratio of mine production to reserves and the market price. This simplicity allows for the user cost model to be adopted for various resources. However, the (country-specific) availability of resources is not solely determined by reserves. Various geological, social, economic, and environmental factors influence resource availability and, consequently, country-specific depletion time.^25,49−53^ Taking these factors into account when estimating country-specific depletion times will enhance the validity of the user cost model.

Regarding the data source for mine production and reserves, we used the USGS data because it provides both reserves and mine production data for various mineral resources at the country level and is updated every year. However, other sources are also available, and regarding a specific mineral resource, detailed analysis is often available. We calculated user cost-based CFs using mine production data from the British Geological Survey (BGS), which is another main source for mining data as well as the USGS (Figure S15).^54^ Some abiotic resources, including Cu, Ni, Pd, Pt, Ag, Sn, and fossil fuels, showed no difference between the USGS and BGS, while differences were observed for some abiotic resources, such as Fe, Mn, Nb, Ta, and W. Since fossil fuels showed no difference, global external costs of resource use calculated by the BGS data were similar to those calculated by the USGS (Figure S16). However, excluding fossil fuels, the global external cost calculated by the BGS data was approximately 15% larger than those by the USGS, and Fe exhibited the largest share in the global external costs calculated by the BGS (Figure S16). This is mainly due to the difference in the mine production of iron ore in China in 2020 (USGS: 360 million tons; BGS: 845 million tons). These results suggest that the data source would be a source of uncertainty for calculating the user cost. In addition, existing studies exploring natural stock estimates for a specific resource, such as copper, suggested that coverage and accuracy of national estimates (e.g., USGS) would be incomplete and reserves data involve uncertainty.^55,56^ Coverage of the USGS data is one of the limitations in this study since the USGS provides no data for some countries and metals. Especially, the USGS provided reserve data of Tantalum for only two countries in 2020. Furthermore, as mentioned in the Section 2, the proved (1P) reserves for oil were adopted in this study while the use of oil consultancy proved-plus-probable (2P) reserves was recommended.^34^ Although country-specific 2P reserves for oil are not available in free databases and estimation may require some costs and efforts, updating the reserves for oil is significant given that oil is one of the most significant resources in terms of global external costs (Figure 4). For example, the estimates of 1P and 2P oil reserves in 2016 suggested that 2P reserves were about 70% larger than 1P reserves.^57^ This means that the calculated user cost for oil can decrease by about 80% if the mine production and market price do not change. Improving the natural stock estimates and exploring uncertainties of the natural stocks and sensitivities of results are important challenges in developing characterization models based on the user cost.

This study calculated the CFs based on the current situation, that is, current mine production, reserves, and market prices. However, it is important to acknowledge that mine production can vary in the future due to changes in resource demand and supply of secondary resources (i.e., recycling), leading to changes in CFs for abiotic resources.^45,58^ Particularly, the demand for resources needed for decarbonization technologies is expected to drastically increase.^59^ Additionally, focusing on a specific mining site means mine production will not be constant in the future. For example, decreasing the reserves (i.e., increasing scarcity) may reduce annual mine production, leading to an increase in the depletion time. We should recognize that the user cost-based CFs in this study are based on various assumptions and limited conditions. Market prices of resources can also fluctuate due to various factors, such as resource demand, ore grade, and technological advancements, which can influence reserves.^32,60,61^ Forecasting these parameters, especially market prices and reserves, is highly uncertain and thus challenging.^62,63^ Nevertheless, assessing future external costs associated with resource use based on future user costs by considering different scenarios and accounting for uncertainties can provide valuable insights into future-oriented resource management.

The dissipation of abiotic resources has been a subject of discussion in LCA.^17,64,65^ To quantify the impacts of dissipation in a product system in LCA, it is necessary to estimate dissipative flows in life cycle inventory as well as assess their impact or loss of resource value in LCIA.^66−69^ Although dissipation is an issue not only at mining sites but also throughout the life cycle of abiotic resources, one way to adopt the user cost model for dissipation may be using the weighted average of CFs (i.e., WCF) to assess the impact of dissipative flows. Integrating dynamic material flow analysis to estimate the dissipation and lifetime of abiotic resources with the user cost concept has the potential to contribute to the assessment of external costs of dissipation.^70,71^ However, defining the depletion time can be an issue in adopting the user cost for assessing the impact of dissipative flow. Since dissipation focuses not on the depletion of natural stocks but on the dilution of total stocks, depletion time should be defined in different ways. For example, Charpentier Poncelet et al.^70^ estimated the average lifetime of metals in the economy by considering the loss of metals over their life cycle and provided the loss rate as the inverse function of the average lifetime. Given that the loss of metals in a specific year originates from not only metal extraction in the year but also the life cycle of metals extracted before the year, dissipation can be estimated by multiplying the anthropogenic stock by the loss rate. For sensitivity analysis, we updated depletion time by replacing the mine production with dissipation and reserves with total stock (reserves plus anthropogenic stock) and calculated user cost-based CFs with the updated depletion time for the six metals (Al, Cu, Fe, Pb, Ni, and Zn) in 2010 based on previous studies (Table S5).^70,72^ The results suggest that the CFs can substantially decrease due to the longer depletion time when dissipation and anthropogenic stocks are considered. However, the increase in the depletion time differed among metals, which means that the relative values of CFs can change among metals, and thus, the decision-making based on the user cost will be influenced. The loss rate had a great effect on the updated depletion time: the lower loss rate, which is mainly attributed to the higher yield ratio and recycling rate and longer lifetime of the final products, resulted in the longer depletion time and lower CF, as observed in Fe and Al. It should be noted that the depletion time and CFs for the fossil fuels will not change because they can be assumed to have no anthropogenic stock (i.e., dissipative), which suggests that the share of the fossil fuels in the global external cost can be higher if the same concept of external cost is adopted for both metals and fossil fuels. Finally, it should be noted that the criterion of dissipation is difficult to determine and can change over time.^17^ Dissipation does not necessarily mean the loss of forever. Technologies that allow extraction and recycling with higher cost or from lower grade deposits could change dissipative flows to nondissipative flows and consequently depletion time. Continuous discussion for adopting the characterization model, including the user cost concept, in assessing dissipation would be necessary.

Country-specific assessment is one of the characteristics of the characterization models based on the user costs presented in this study. The results suggest that the external costs of resource use vary depending on the mining country (Figure 2). However, note that these results do not necessarily suggest that we should avoid dependence on mining activities in countries with higher external costs (user costs). For sustainable resource mining and use, not only supply chain management but also supporting mining sites and mine development by consuming countries would be significant. The country-specific CFs based on user cost will support resource management focusing on mining countries. Our results revealed mining countries with higher external costs associated with resource mining and hotspot resources in terms of external costs (Figure S3). We could explore effective measures to alleviate the external costs in these countries (e.g., new deposit exploration) through international cooperation for achieving sustainable resource use. For such a discussion, country-specific characterization factors can be used to discuss the responsibility of consuming countries in terms of inducing external costs by incorporating material footprint (consumption-based accounting) studies.^27,73^ Therefore, the application of the proposed country-specific CFs can be interpreted as oriented toward sustainable mining, considering the diversity of reserves. However, in this sense, we need to continue the discussion on whether the application of the country-specific CFs is within the scope of conventional LCA or beyond it, such as criticality assessment and life cycle sustainability assessment.

## Supporting Information Available

List of data sources; additional results for external costs of abiotic resource use; additional results for time-series clustering; sensitivity analysis; and user cost considering anthropogenic stock and dissipation (PDF)
List of characterization factors; data for mine production, reserves, and price; and comparison with other methods (XLSX)
