# Supplementary material for: Country-Specific External Costs of Abiotic Resource Use Based on User Cost Model in Life Cycle Impact Assessment
Source: Environ Sci Technol. 2024 Apr 26;58(18):7849–59. doi: 10.1021/acs.est.4c00100 (PMC11080043; doi:10.1021/acs.est.4c00100)
Supplement: Supplementary file 1 — es4c00100_si_001.pdf [file es4c00100_si_001.pdf]

Supporting Information

Country-Specific External Costs of Abiotic Resource Use Based on User Cost Model in Life Cycle Impact Assessment

Ryosuke Yokoi[a,*], Masaharu Motoshita[a], Takeshi Matsuda[b], Norihiro Itsubo[c]

[a] Research Institute of Science for Safety and Sustainability, National Institute of Advanced Industrial Science and Technology (AIST), 16-1 Onogawa, Tsukuba, Japan

[b] Pacific Power Co., Ltd., 3-22 Kandanishikicho, Chiyoda, Tokyo, Japan

[c] Faculty of Science and Engineering, Waseda University, 3-4-1 Okubo Shinjuku-ku, Tokyo, Japan

* Corresponding author

Summary: 22 pages, 5 tables, 16 figures.

Table S1. Data sources of mine production, reserves, and market price in 2020.

	Mine production	Reserves	Market price
Aluminum	USGS[1]	USGS[1]	USGS[4], USGS[5]
Antimony	USGS[1]	USGS[1]	USGS[4], USGS[5]
Barium	USGS[1]	USGS[1]	USGS[4], USGS[5]
Boron	USGS[1]	USGS[1]	USGS[4], USGS[5]
Chromium	USGS[1]	USGS[1]	USGS[4], USGS[5]
Cobalt	USGS[1]	USGS[1]	USGS[4], USGS[5]
Copper	USGS[1]	USGS[1]	USGS[4], USGS[5]
Fluorine	USGS[1]	USGS[1]	USGS[4], USGS[5]
Gold	USGS[1]	USGS[1]	USGS[4], USGS[5]
Iron	USGS[1]	USGS[1]	USGS[4], USGS[5]
Lead	USGS[1]	USGS[1]	USGS[4], USGS[5]
Lithium	USGS[1]	USGS[1]	USGS[4], USGS[5]
Magnesium	USGS[1]	USGS[1]	USGS[4], USGS[5]
Manganese	USGS[1]	USGS[1]	USGS[4], USGS[5]
Molybdenum	USGS[1]	USGS[1]	USGS[4], USGS[5]
Nickel	USGS[1]	USGS[1]	USGS[4], USGS[5]
Niobium	USGS[1]	USGS[1]	USGS[4], USGS[5]
Palladium	USGS[1]	USGS[1]	USGS[4], USGS[5]
Phosphorus	USGS[1]	USGS[1]	USGS[4], USGS[5]
Platinum	USGS[1]	USGS[1]	USGS[4], USGS[5]
Rhenium	USGS[1]	USGS[1]	USGS[4], USGS[5]
Silver	USGS[1]	USGS[1]	USGS[4], USGS[5]
Tantalum	USGS[1]	USGS[1]	USGS[4], USGS[5]
Tin	USGS[1]	USGS[1]	USGS[4], USGS[5]
Titanium	USGS[1]	USGS[1]	USGS[4], USGS[5]
Tungsten	USGS[1]	USGS[1]	USGS[4], USGS[5]
Uranium	NEA[2]	NEA[2]	Trading Economist[6]
Vanadium	USGS[1]	USGS[1]	USGS[4], USGS[5]
Zinc	USGS[1]	USGS[1]	USGS[4], USGS[5]
Coal	Energy Institute[3]	Energy Institute[3]	Energy Institute[3]
Natural gas	Energy Institute[3]	Energy Institute[3]	Energy Institute[3]
Oil	Energy Institute[3]	Energy Institute[3]	Energy Institute[3]

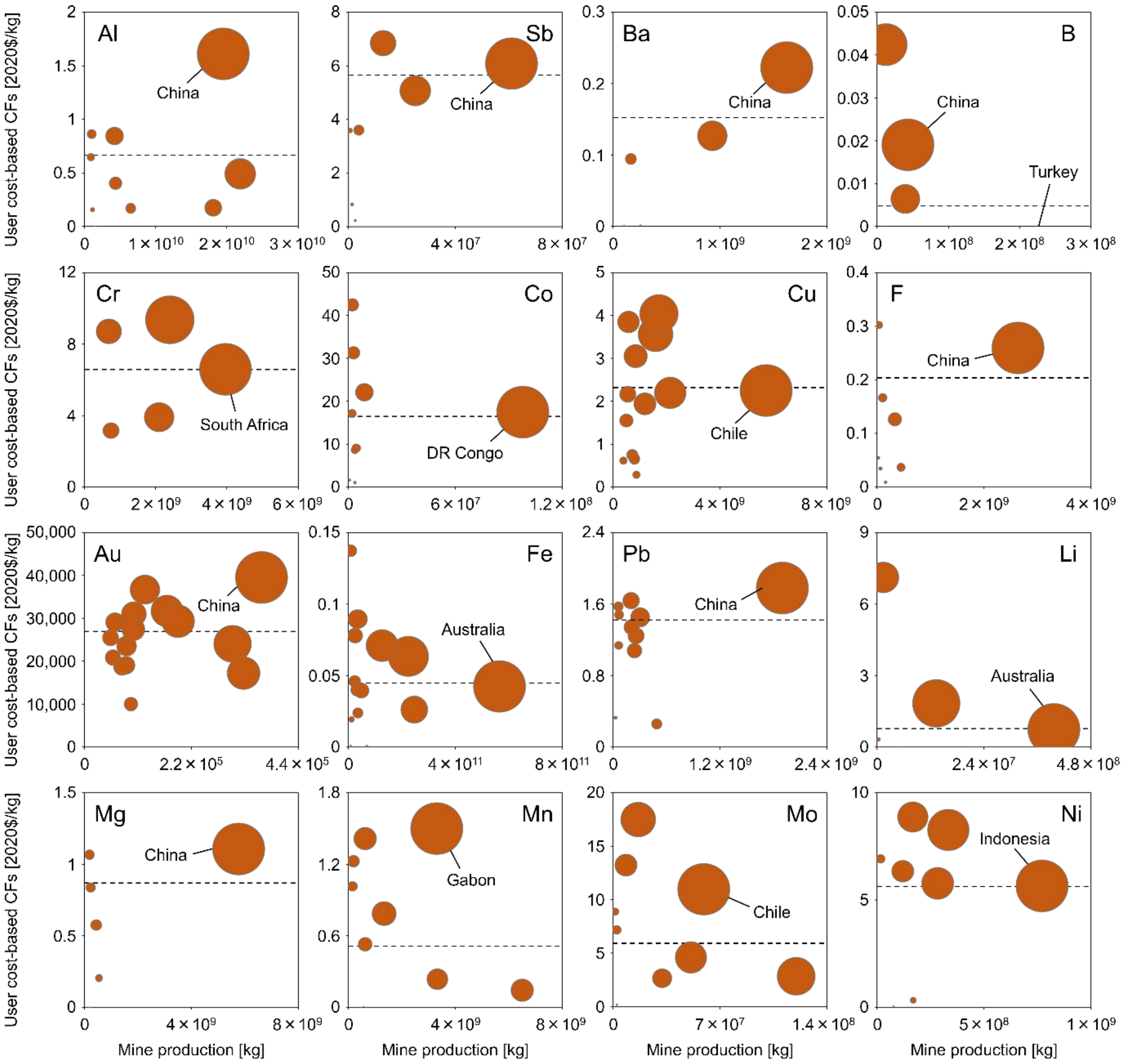

Figure S1. Relationships between mine production and characterization factors (CFs) based on user cost in mining countries in 2020. The size of the circles represents the user cost, which is calculated by multiplying the mine production and CFs. The weighted average of CFs (WCF) is shown by dotted lines.

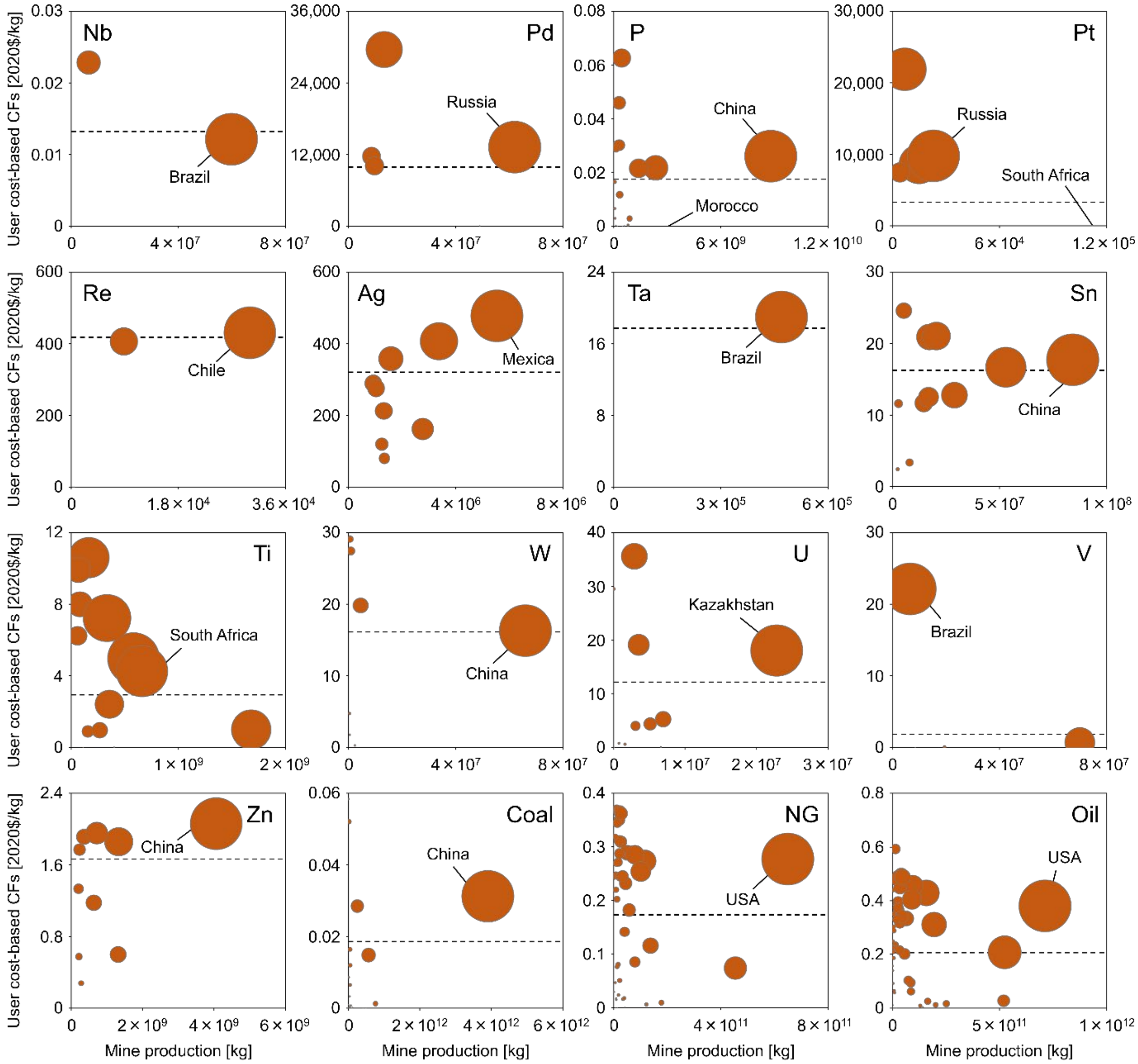

Figure S1. (continued).

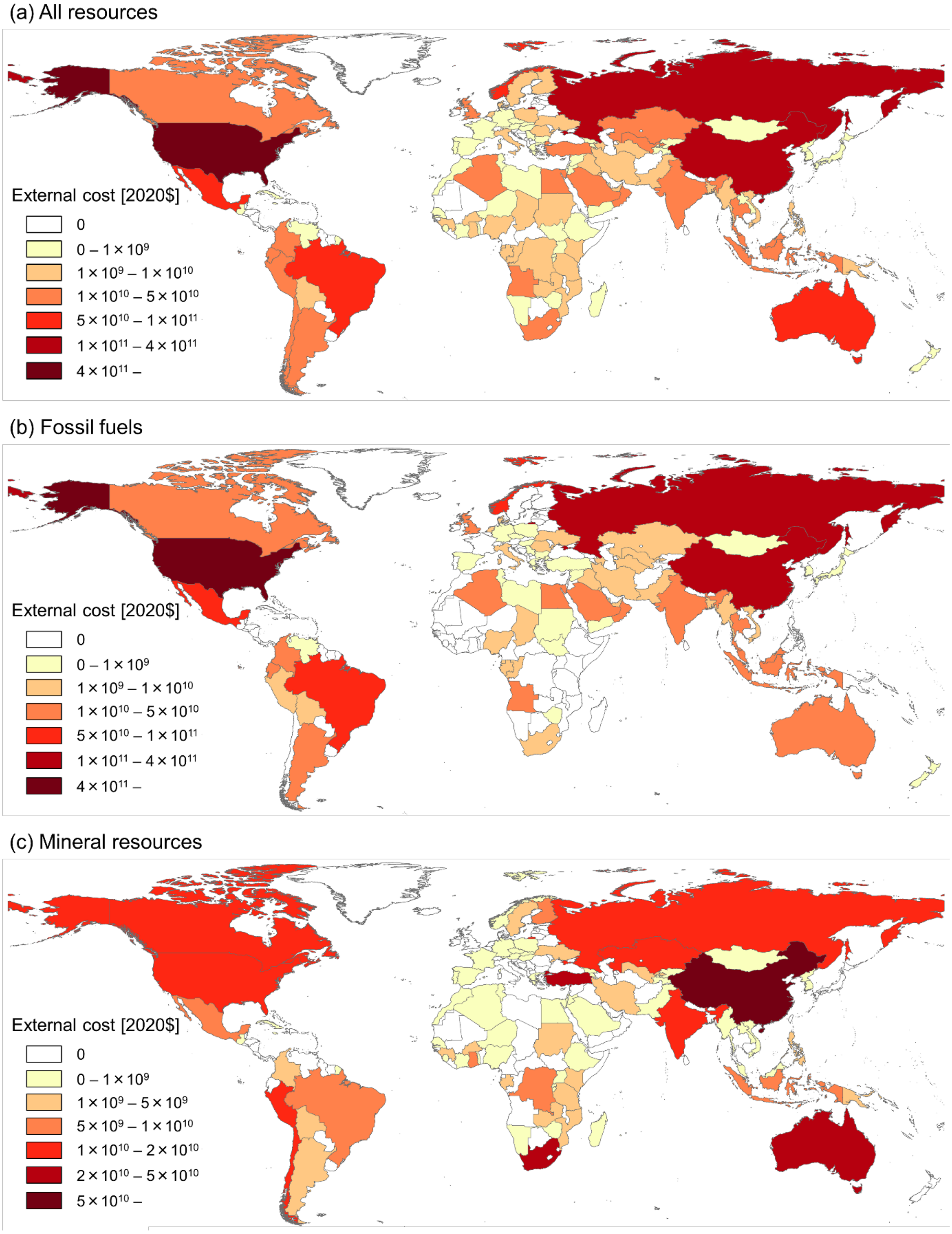

Figure S2. Distribution of external costs of resource use in 2020.

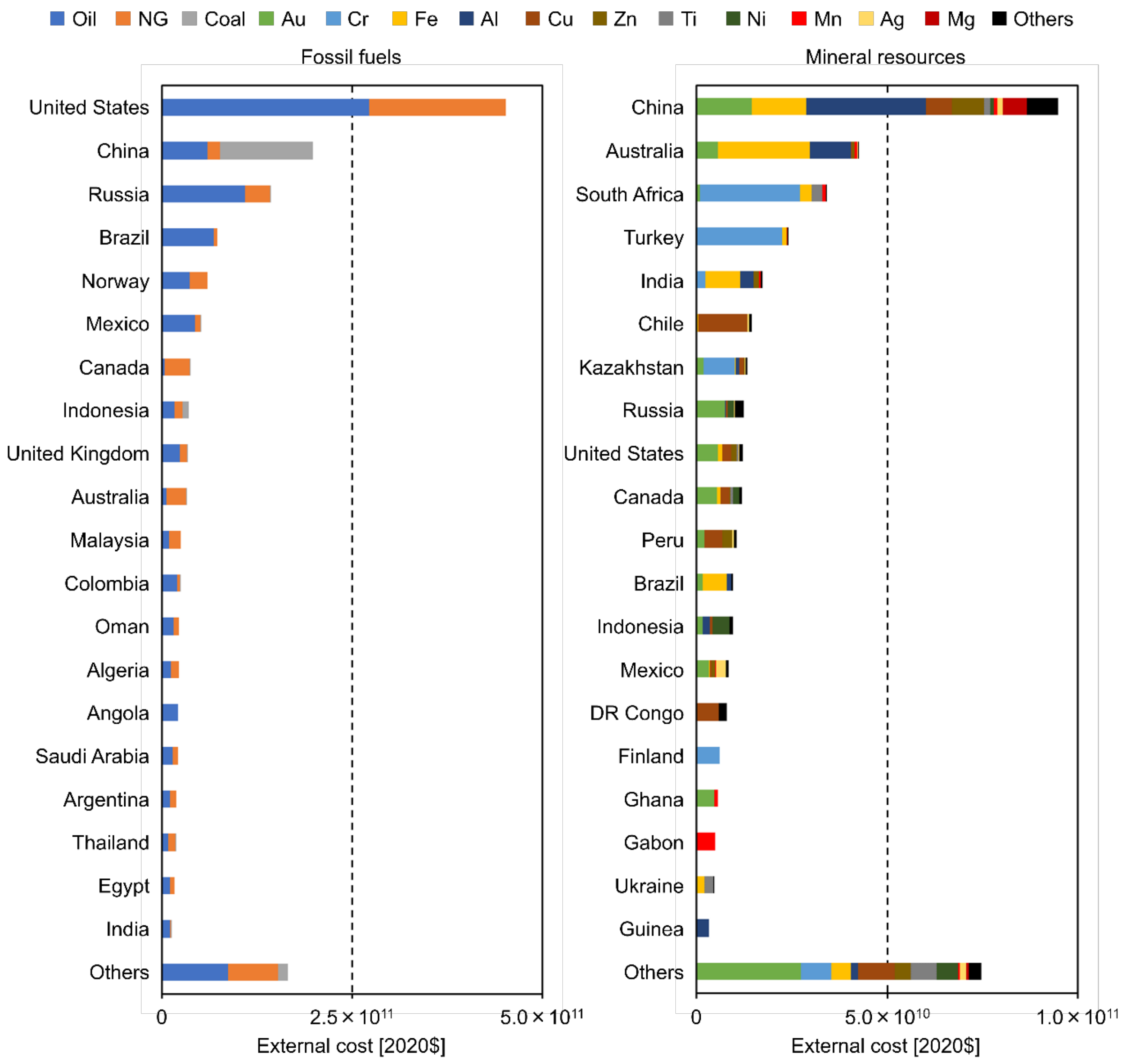

Figure S3. External costs of fossil fuels and mineral resources for countries with highest external costs in 2020.

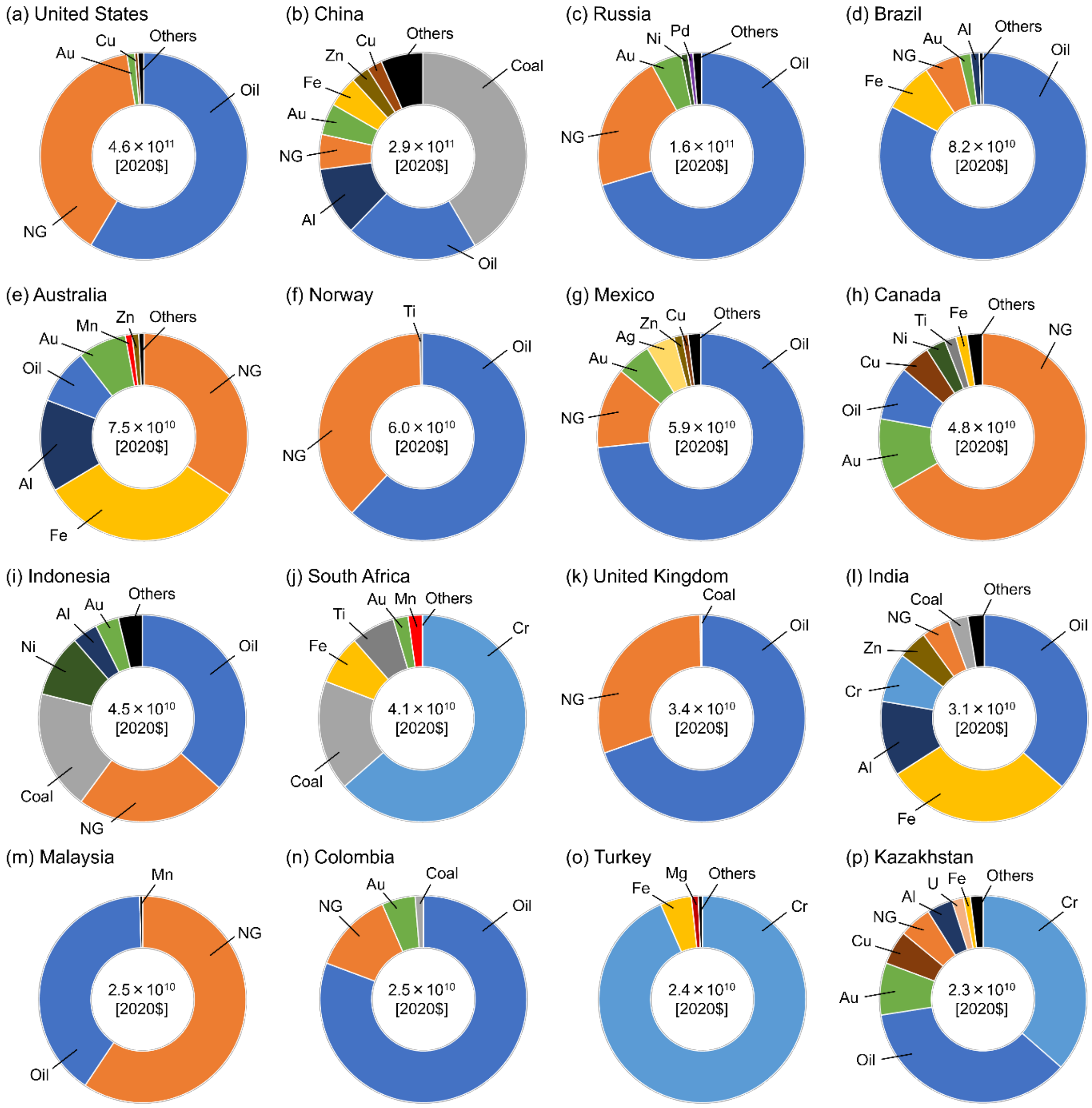

Figure S4. Share of external costs of resources for countries with highest external costs in 2020.

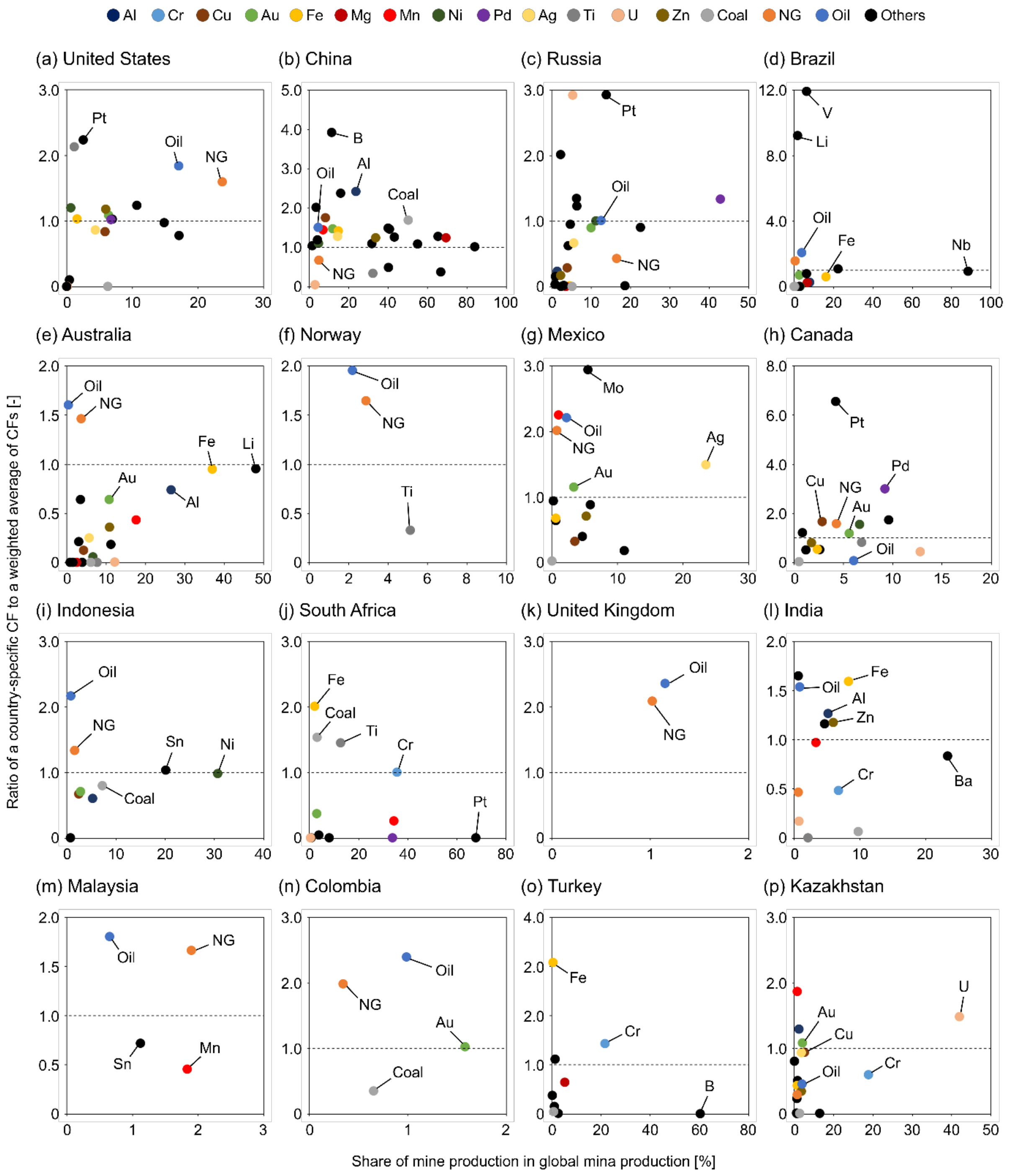

Figure S5. Ratio of a country-specific CF to a weighted average of CFs (WCF) and share of mine production in global mine production for countries with highest external costs in 2020.

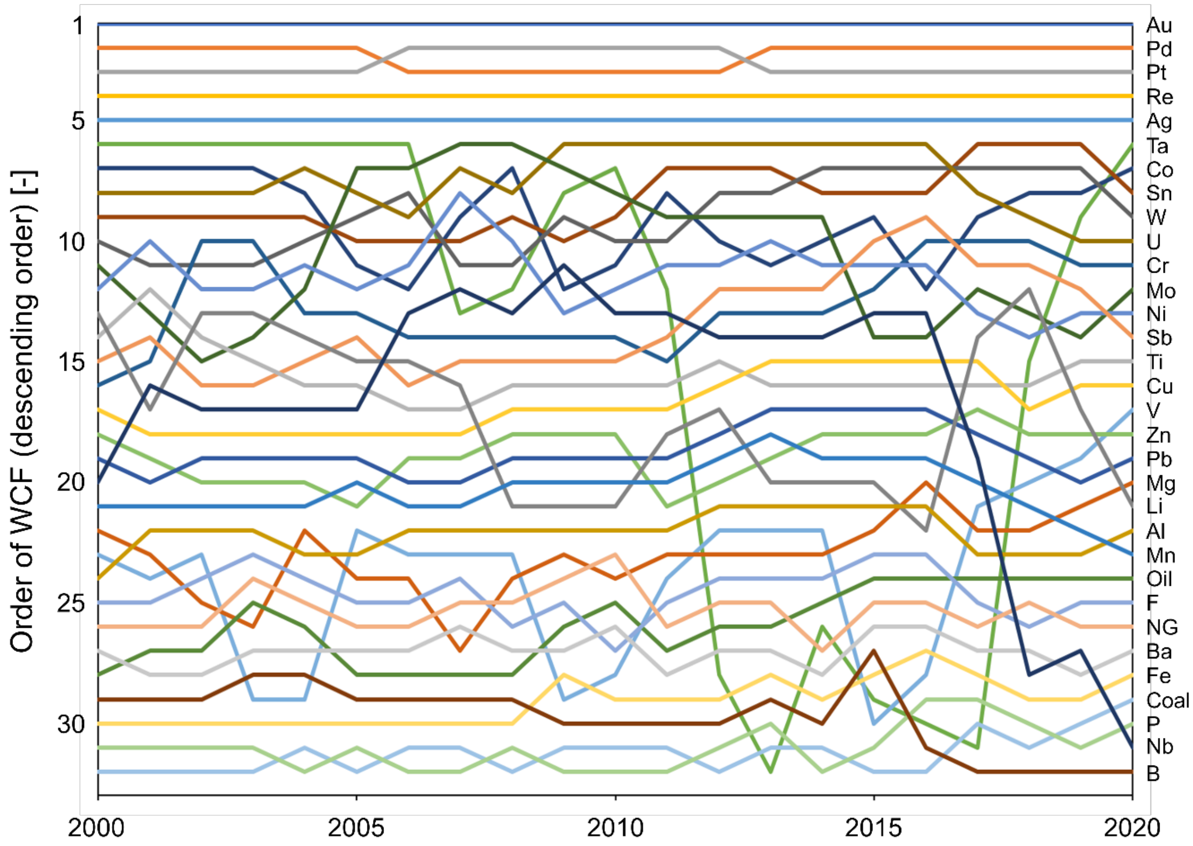

Figure S6. Transition of the order of the WCF for the target resources (descending order).

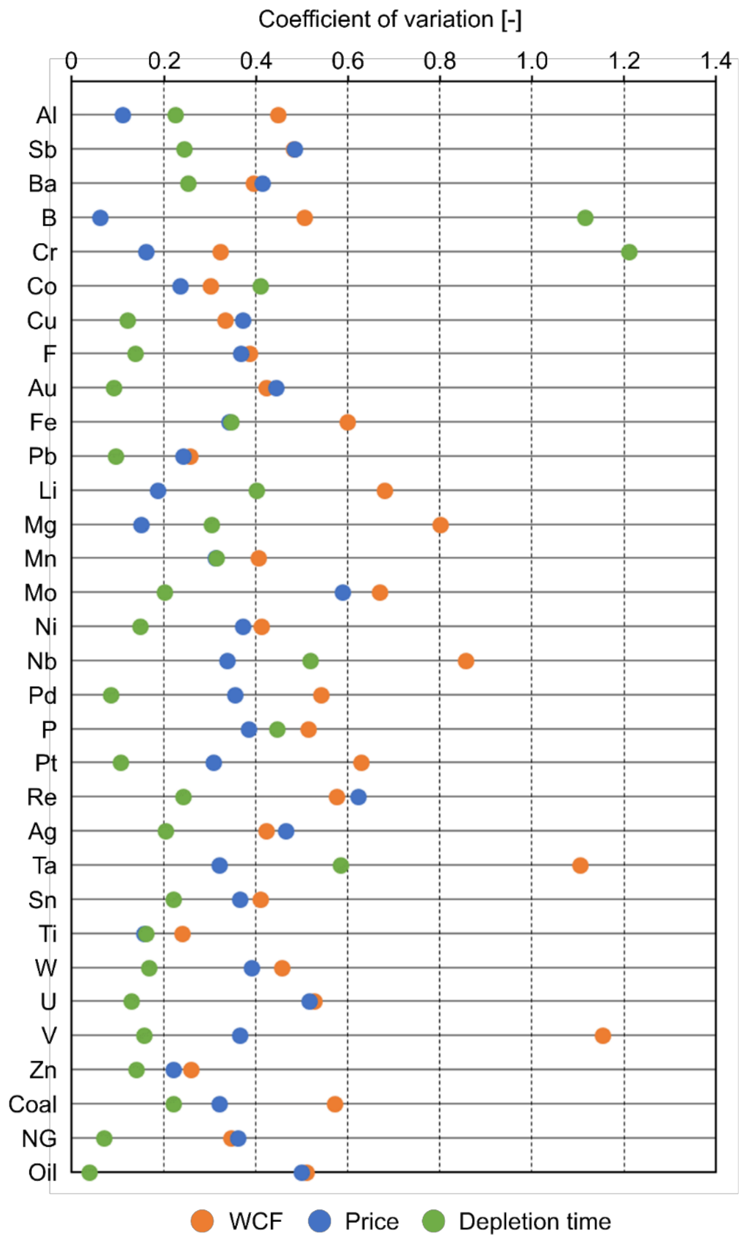

Figure S7. Coefficient of variation of the weighted average of CFs based on user cost, price, and depletion time.

Table S2. The Pearson correlation coefficient for historical data for price and WCF, and depletion time and WCF.

	The Pearson correlation coefficient	
	Price and WCF	Depletion time and WCF
Al	0.29	-0.87
Sb	1.00	-0.59
Ba	0.98	0.63
B	-0.13	-0.89
Cr	0.64	-0.83
Co	0.74	-0.32
Cu	0.98	0.62
F	0.97	-0.39
Au	1.00	0.05
Fe	0.92	-0.88
Pb	0.99	-0.83
Li	-0.00	-0.63
Mg	0.17	-0.72
Mn	0.90	-0.59
Mo	0.99	-0.80
Ni	0.98	-0.12
Nb	0.61	-0.87
Pd	0.71	-0.32
P	0.87	0.59
Pt	0.89	-0.00
Re	0.96	-0.40
Ag	0.99	0.75
Ta	0.15	-0.52
Sn	0.99	-0.83
Ti	0.77	-0.39
W	0.95	-0.64
U	0.85	0.01
V	0.23	0.01
Zn	0.99	-0.67
Coal	0.74	-0.81
NG	1.00	-0.82
Oil	1.00	0.19

Table S3. Classification of resources based on time-series clustering of price, depletion time, and WCF.

	Price	Depletion time	WCF
Al	P2	D1	W3
Sb	P3	D1	W3
Ba	P3	D3	W3
B	P2	D3	W1
Cr	P2	D1	W3
Co	P1	D1	W1
Cu	P3	D3	W3
F	P3	D2	W3
Au	P3	D2	W3
Fe	P3	D1	W2
Pb	P3	D2	W3
Li	P3	D1	W3
Mg	P3	D1	W3
Mn	P2	D2	W2
Mo	P2	D2	W2
Ni	P2	D1	W2
Nb	P2	D2	W2
Pd	P3	D1	W3
P	P3	D3	W2
Pt	P2	D2	W2
Re	P2	D1	W2
Ag	P3	D3	W2
Ta	P1	D1	W1
Sn	P3	D1	W3
Ti	P2	D1	W2
W	P3	D1	W3
U	P2	D3	W2
V	P2	D2	W3
Zn	P3	D2	W3
Coal	P3	D1	W3
NG	P3	D1	W3
Oil	P3	D2	W3

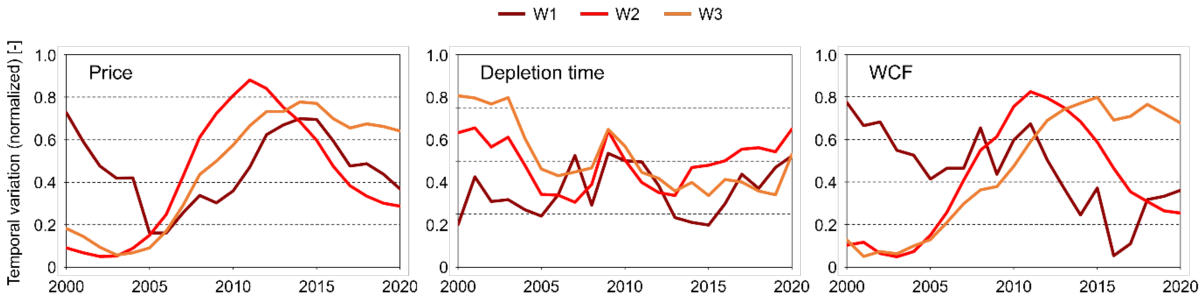

Figure S8. Average historical trends of price, depletion time, and WCF for the clusters of WCF. Historical trends are normalized in the range 0–1.

Table S4. Minimum and maximum values in the normalized average historical trends of price, depletion time, and WCF for each cluster.

		Minimum		Maximum		Maximum/Minimum
		Year	Value	Year	Value	
Price	P1	2005	0.13	2000	0.92	6.94
	P2	2003	0.08	2011	0.81	10.56
	P3	2003	0.05	2014	0.83	15.40
Depletion time	D1	2019	0.12	2001	0.70	5.63
	D2	2013	0.25	2000	0.63	2.49
	D3	2000	0.10	2020	0.90	8.68
WCF	W1	2016	0.05	2000	0.77	14.34
	W2	2003	0.05	2011	0.83	16.49
	W3	2001	0.05	2015	0.80	15.85

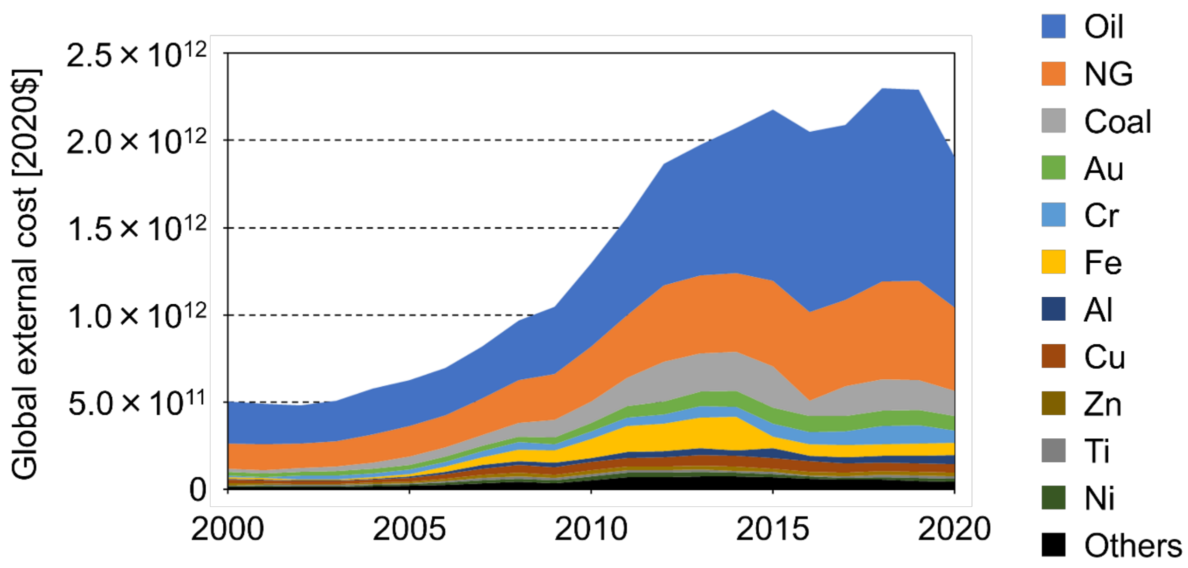

Figure S9. Historical global external costs of resource use based on the user cost.

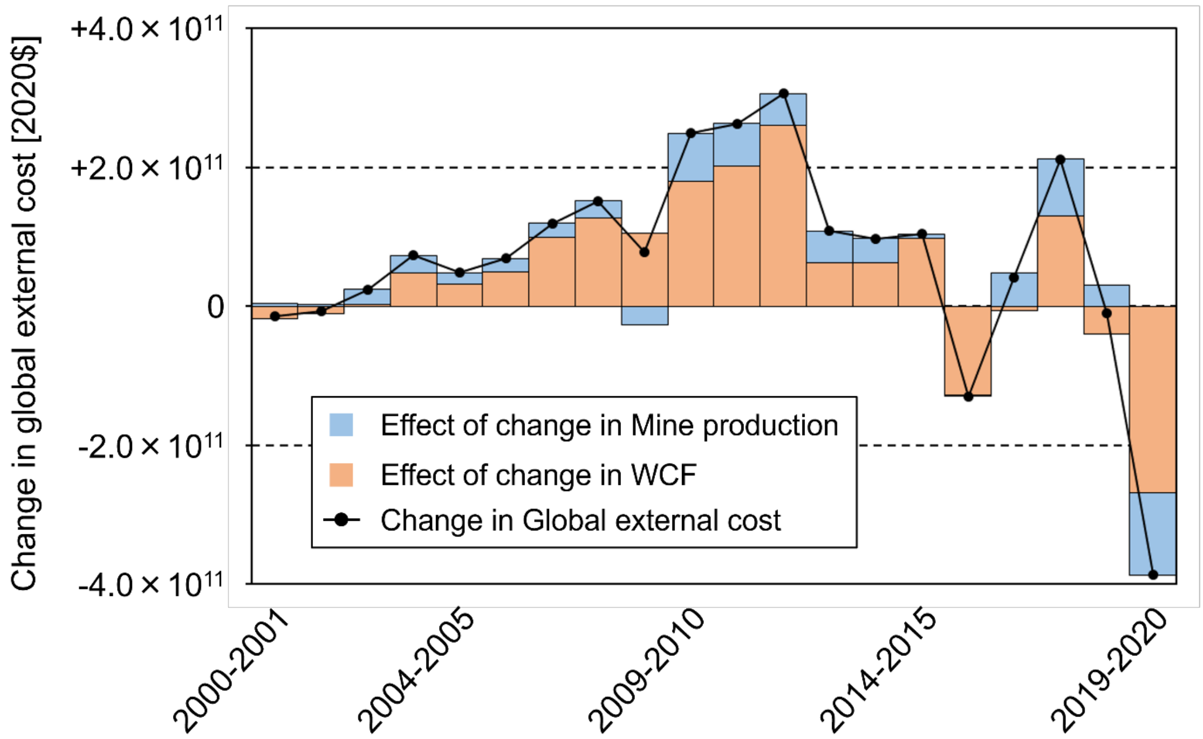

Figure S10. Effects of changes in mine production and WCF on the change in global external cost of resource use. The logarithmic mean divisia index (LMDI) approach (additive) was adopted for decomposition analysis.

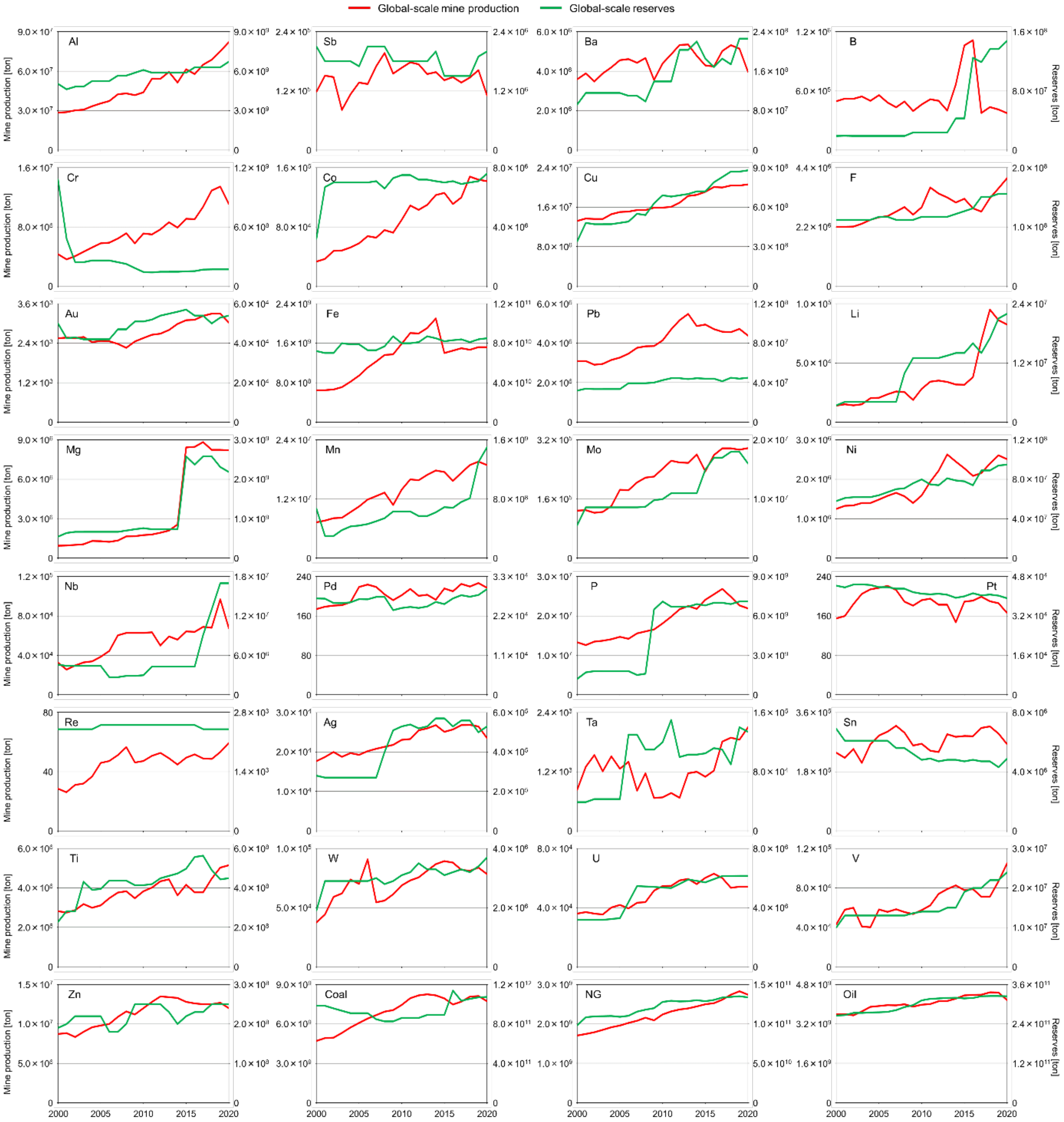

Figure S11. Historical trends of global-scale mine production and reserves.

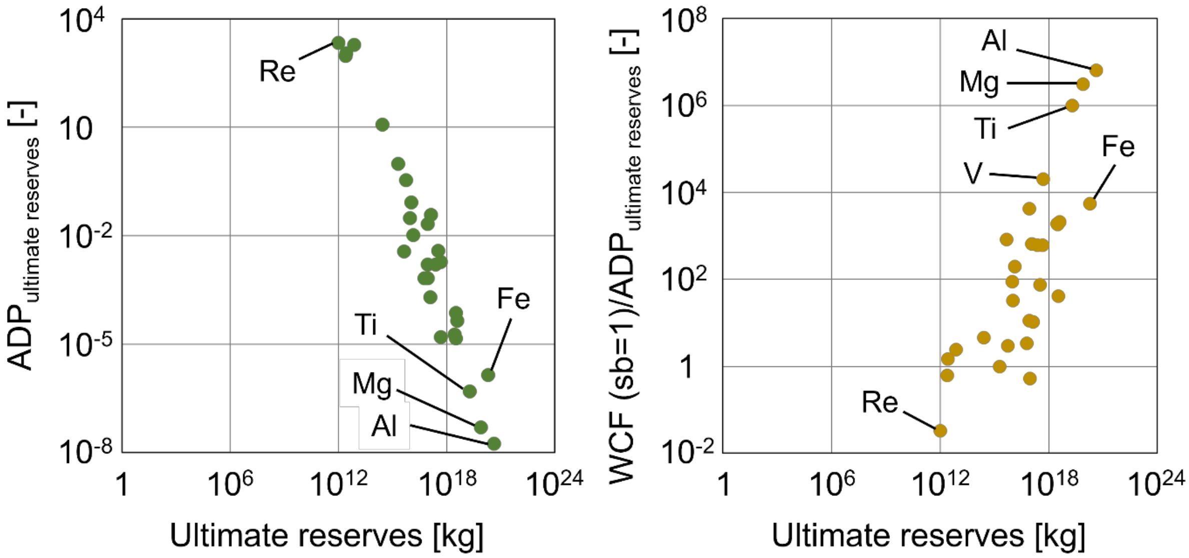

Figure S12. Relationships between the ultimate reserves, ADP, and weighted average of CFs.

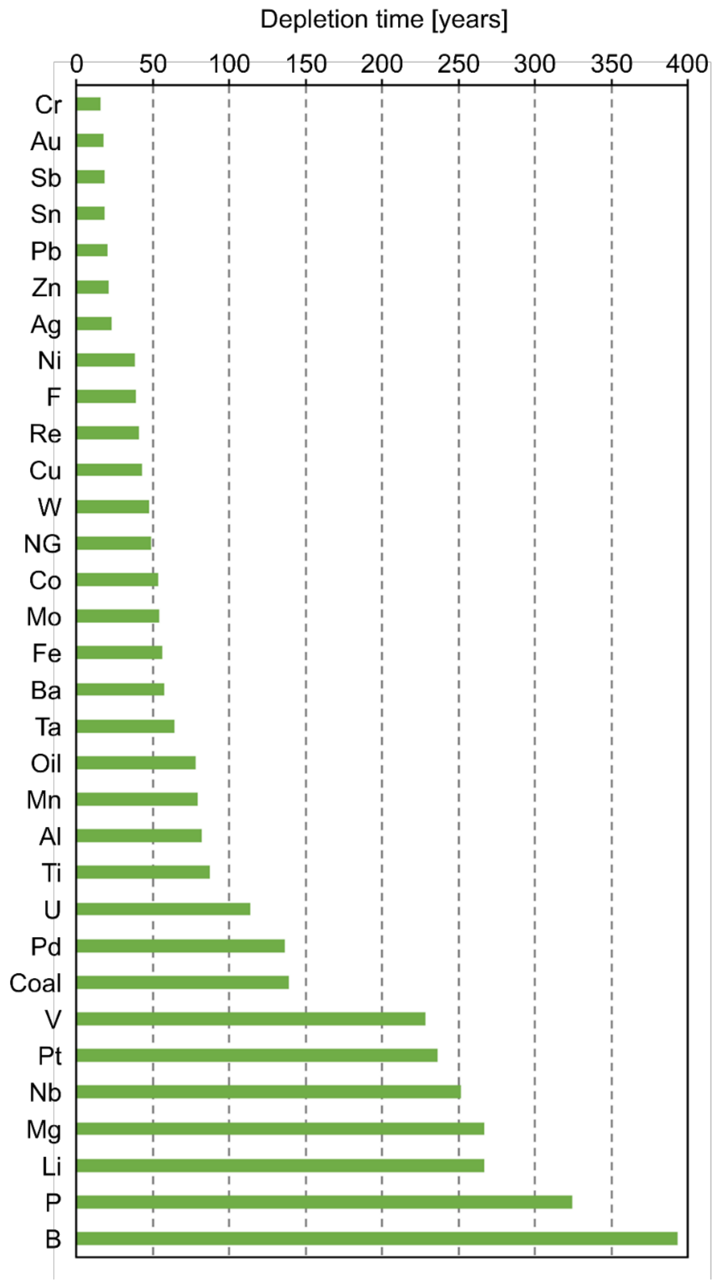

Figure S13. Global-scale depletion time of the target resources in 2020 (ascending order).

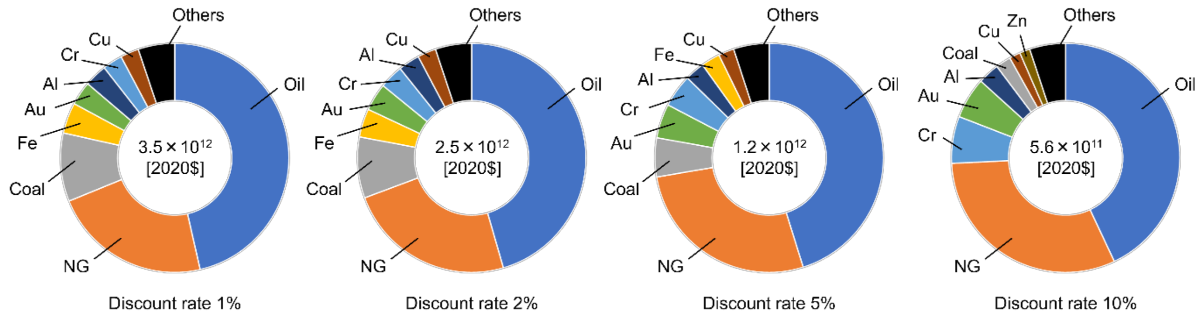

Figure S14. Global external costs of resource use based on user cost with different discount rates in 2020.

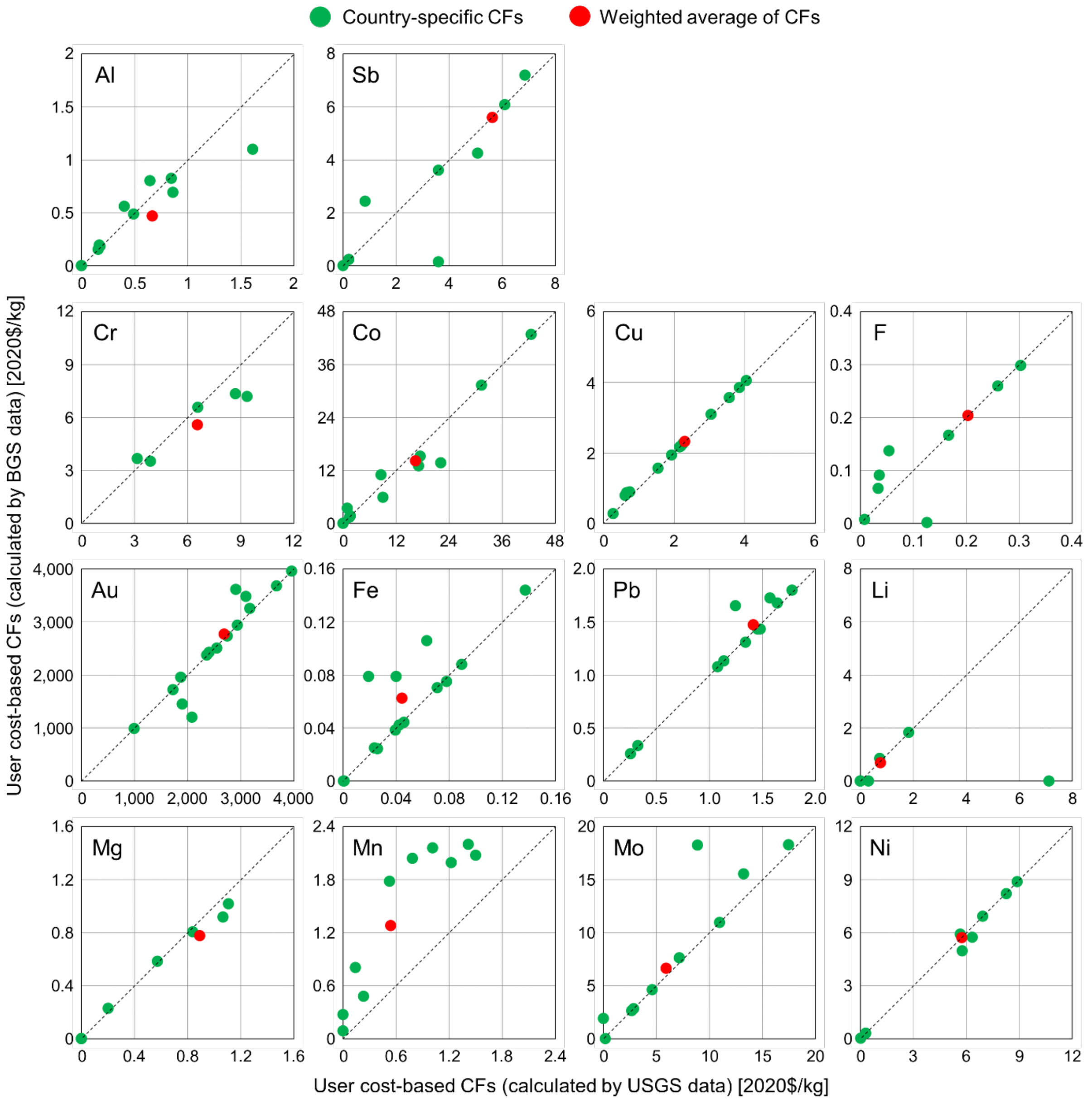

Figure S15. Comparisons between user cost-based characterization factors calculated by USGS and BGS mine production data in 2020. USGS data for reserves is used for both calculations. Barium and boron are excluded because BGS do not provide data of mine production for these resources.

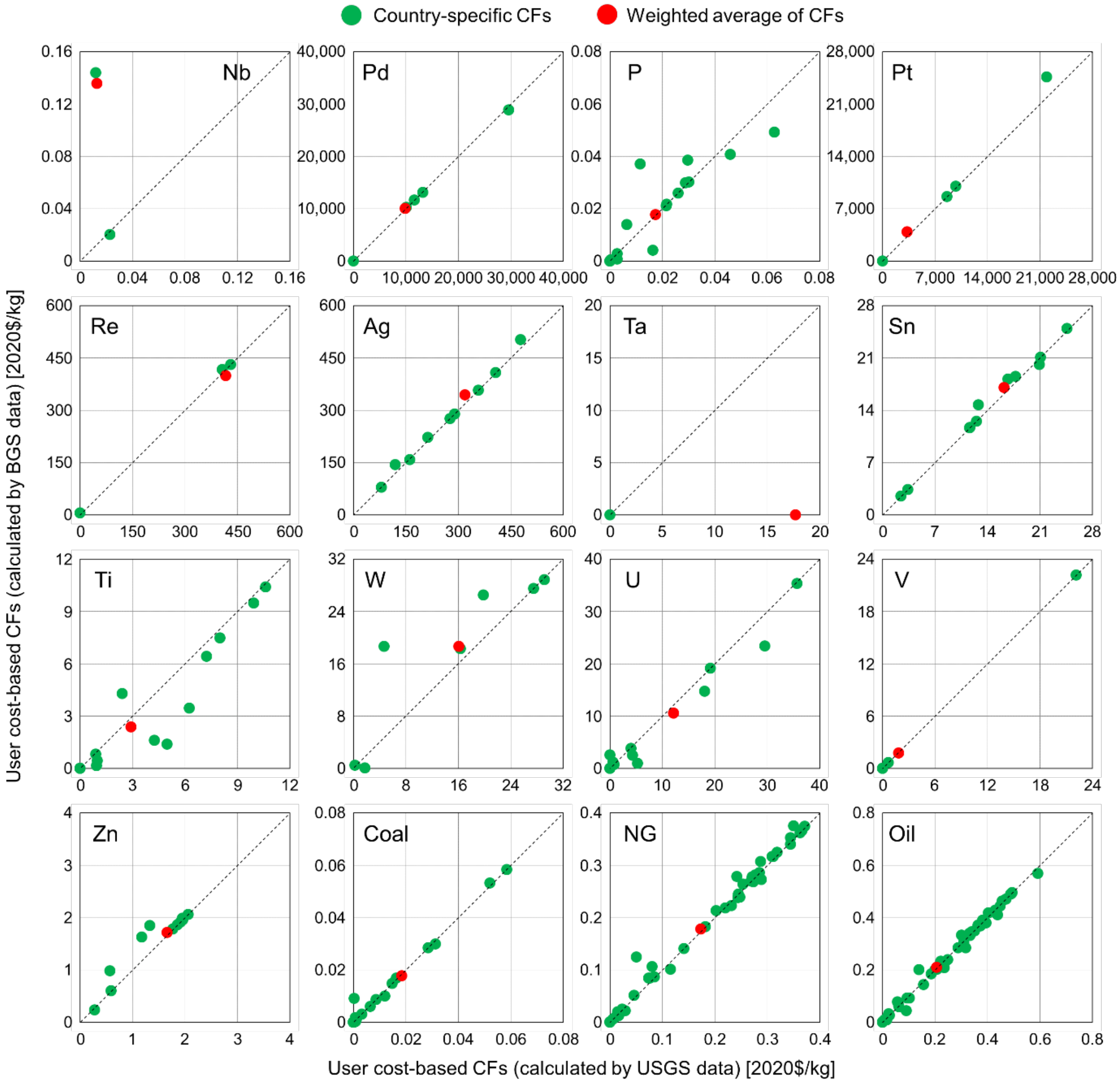

Figure S15. (continued).

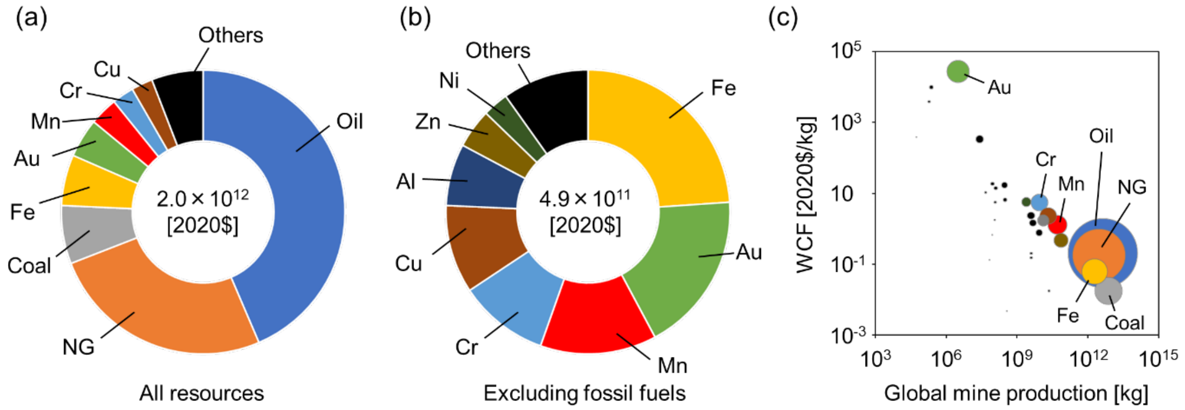

Figure S16. Share of global external costs of resource use based on the user cost by BGS data in 2020. (a) All target abiotic resources. (b) Target abiotic resources excluding fossil fuels. (c) Relationships between global mine production and WCF of resources. The size of the circles represents the global external costs (i.e., the product of global mine production and WCF). The colors of the circles correspond to those in (a) and (b). USGS data are adopted for calculating global external costs of barium and boron.

Table S5. User cost-based characterization factors (CFs) considering the anthropogenic stock and dissipation for the six metals in 2010. Data for mine production, reserves, and anthropogenic stock is global scale.

	Mine production (10^3 ton)	Reserves (10^3 ton)	Anthropogenic stock* (10^3 ton)	Loss rate (kg loss/year/kg)	Dissipation** (10^3 ton /year)	Original depletion time (year)	Updated depletion time*** (year)	5-year price (2020$/kg)	Original user cost-based CFs (2020$/kg)	Updated user cost-based CFs (2020$/kg)
Al	43,890	5,880,000	692,572	0.013	9,144	134	719	3.00	0.0571	1.78×10^{-9}
Cu	15,900	630,000	295,755	0.022	6,510	40	142	8.47	2.62	0.127
Fe	1,218,824	87,000,000	24,422,795	0.006	158,676	71	702	0.162	0.0197	1.57×10^{-10}
Pb	4,140	80,000	53,489	0.037	1,962	19	68	2.82	1.59	0.377
Ni	1,590	76,000	28,274	0.017	483	48	216	29.6	7.20	5.02×10^{-2}
Zn	12,000	250,000	135,689	0.040	5,472	21	70	3.20	1.73	0.392
Ref.	[7]	[8]	[9]	[10]						

*Anthropogenic stock represents metal stock used in society in the form of final products.

**Dissipation is calculated by multiplying the anthropogenic stock by the loss rate.

***Updated depletion time is calculated by dividing the total stock (reserves plus anthropogenic stock) by dissipation.

References

1. USGS, 2022. Mineral Commodity Summaries 2022. https://doi.org/10.3133/mcs2022.
2. NEA, 2020. Uranium 2020: Resources, Production, and Demand. https://www.oecd-nea.org/jcms/pl_52718/uranium-2020-resources-production-and-demand (accepted 8 December 2022).
3. Energy Institute, Statistical Review of World Energy Data. https://www.energyinst.org/statistical-review/resources-and-data-downloads (accessed 1 November 2023).
4. USGS, Historical Statistics for Mineral and Material Commodities in the United States. https://www.usgs.gov/centers/national-minerals-information-center/historical-statistics-mineral-and-material-commodities (accessed 8 Dec 2022).
5. USGS, 2013. Metal Prices in the United States through 2010: U.S. Geological Survey Scientific Investigations Report 2012–5188. http://pubs.usgs.gov/sir/2012/5188 (accessed 8 Dec 2022).
6. Trading Economics, Uranium. https://tradingeconomics.com/commodity/uranium (accessed 8 Dec 2022).
7. USGS, 2012. Mineral Commodity Summaries 2012. https://doi.org/10.3133/mineral2012.
8. USGS, 2011. Mineral Commodity Summaries 2011. https://doi.org/10.3133/mineral2011.
9. Watari, T.; Yokoi, R. International inequality in in-use metal stocks: What it portends for the future. *Resour. Policy* **2021**, 70, 101968. https://doi.org/10.1016/j.resourpol.2020.101968.
10. Charpentier Poncelet, A.; Helbig, C.; Loubet, P.; Beylot, A.; Muller, S.; Villeneuve, J.; Laratte, B.; Thorenz, A.; Tuma, A.; Sonnemann, G. Losses and Lifetimes of Metals in the Economy. *Nat. Sustain.* **2022**, 5, 717–726. https://doi.org/10.1038/s41893-022-00895-8.
